# Downregulation of kainate receptors regulating GABAergic transmission in amygdala after early life stress is associated with anxiety-like behavior in rodents

**DOI:** 10.1038/s41398-021-01654-7

**Published:** 2021-10-18

**Authors:** Jonas Englund, Joni Haikonen, Vasilii Shteinikov, Shyrley Paola Amarilla, Tsvetomira Atanasova, Alexandra Shintyapina, Maria Ryazantseva, Juha Partanen, Vootele Voikar, Sari E. Lauri

**Affiliations:** 1 Molecular and Integrative Biosciences Research Program, University of Helsinki, Helsinki, Finland; 2 HiLife Neuroscience Center, University of Helsinki, Helsinki, Finland; 3 Department of Veterinary Biosciences, Faculty of Veterinary Medicine, University of Helsinki, Helsinki, Finland

**Keywords:** Neuroscience, Psychiatric disorders

## Abstract

Early life stress (ELS) is a well-characterized risk factor for mood and anxiety disorders. GABAergic microcircuits in the amygdala are critically implicated in anxiety; however, whether their function is altered after ELS is not known. Here we identify a novel mechanism by which kainate receptors (KARs) modulate feedforward inhibition in the lateral amygdala (LA) and show that this mechanism is downregulated after ELS induced by maternal separation (MS). Specifically, we show that in control rats but not after MS, endogenous activity of GluK1 subunit containing KARs disinhibit LA principal neurons during activation of cortical afferents. GluK1 antagonism attenuated excitability of parvalbumin (PV)-expressing interneurons, resulting in loss of PV-dependent inhibitory control and an increase in firing of somatostatin-expressing interneurons. Inactivation of *Grik1* expression locally in the adult amygdala reduced ongoing GABAergic transmission and was sufficient to produce a mild anxiety-like behavioral phenotype. Interestingly, MS and GluK1-dependent phenotypes showed similar gender specificity, being detectable in male but not female rodents. Our data identify a novel KAR-dependent mechanism for cell-type and projection-specific functional modulation of the LA GABAergic microcircuit and suggest that the loss of GluK1 KAR function contributes to anxiogenesis after ELS.

## Introduction

Disturbed activity early in life has lasting effects on the limbic circuitry, which predisposes to pathological behavioral phenotypes later on in life. Early life stress (ELS) correlates with an increased risk of developing mood and anxiety disorders and is associated with altered synaptic plasticity and excitability in the amygdala, a part of the limbic system regulating emotional responses (e.g., [1–3]). Stress-induced changes in the neuronal structure and function of the amygdala have been implicated in anxiogenic behavior both in humans and in rodent models (e.g. [4–6]). However, the detailed molecular and cellular mechanisms underlying these changes remain largely unknown.

Kainate receptors (KARs) represent a family of ionotropic glutamate receptors that regulate neuronal excitability, synaptic transmission and plasticity in various parts of the brain (reviewed by Lerma [7]). In the amygdala, KARs contribute to transmission and associative plasticity at glutamatergic inputs to lateral amygdala (LA) (e.g. [8, 9]) and regulate GABAergic drive in the basal nucleus (BA) [10, 11]. In addition, genetic inactivation or overexpression of KAR subunits (GluK1 and GluK4, respectively) alters transmission efficacy from basolateral amygdala (BLA) to central amygdala [11–13], the circuitry responsible for main amygdala output. KARs are thus perfectly positioned to modulate amygdala circuits implicated in anxiety-like behaviors. Indeed, pharmacological and genetic evidence supports that KARs, and in particular, GluK1 subunit-containing KARs, regulate anxiety-like behaviors in rodents [11, 12, 14–18]. In humans, genes encoding KAR subunits, including *GRIK1* encoding the subunit GluK1, have been implicated in mood disorders ([19–23]; reviewed by Lerma and Marquez [24]).

Here we show that maternal separation (MS) stress early in life leads to downregulation of *Grik1* mRNA expression in the LA, and further, that inactivation of *Grik1* expression locally in the adult amygdala is sufficient to produce a mild anxiety-like behavioral phenotype in rodents. Interestingly, both phenomena were predominant in males, similar to the anxiogenic behavior induced by MS. Ex vivo investigation of the LA circuit function in rats that had experienced MS revealed significant changes in KAR-dependent regulation of GABAergic microcircuitry responding to cortical activity. Our data identify ELS-dependent modulation of KARs as a novel mechanism for regulation of amygdala GABAergic microcircuits implicated in anxiety behaviors.

## Results

### Maternal separation leads to loss of *Grik1* expression in the lateral amygdala

**Fig. 1:**
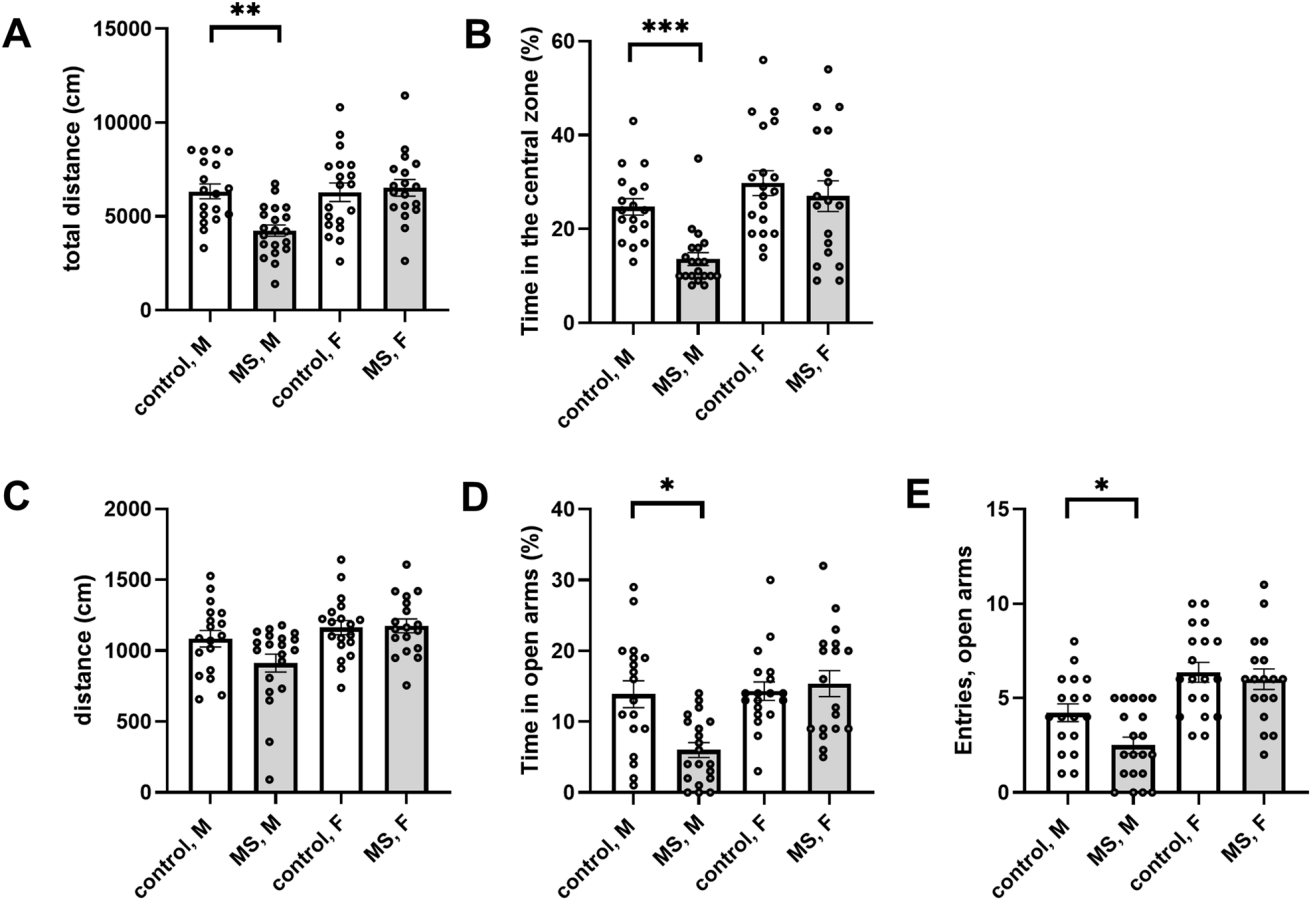
Maternal separation induces anxiety-like behavior. **A** Total distance traveled in the open field test for male (M) and female (F) rats that have experienced MS early in life (M, *n* = 18; F, *n* = 20) and their littermate controls (M, *n* = 20; F, *n* = 18). There was a significant effect of MS (*F*_(1, 71)_ = 5.10, *p* = 0.03) and gender (*F*_(1, 71)_ = 7.42, *p* = 0.008) on the total distance traveled, and significant interaction effect between MS and gender (*F*_(1, 71)_ = 8.03, *p* = 0.006). Post-hoc comparison revealed that the effect of MS was significant in males (control 6335 ± 380 cm, MS 4245 ± 292 cm; *p < 0.003, Holm–Sidak) but not in females (control 6290 ± 477 cm, MS 6526 ± 433 cm). **B** Percentage of time spent in the central zone of the open field arena in the same test was significantly affected by MS (*F*_(1, 71)_ = 9.997, *p* = 0.002) and there was a significant interaction effect between MS and gender (*F*_(1, 71)_ = 17.57, *p* < 0.0001). Pairwise comparison indicated significant effect of the MS treatment in males (control 27.8 ± 3.6 %, MS 8.0 ± 1.8 %, ***p < 0.0001, Mann–Whitney) but not in females (control 19.0 ± 2.2 %, MS 21.8 ± 2.7 %). **C** Distance traveled during the 5 min testing in the elevated plus maze (EPM) for the MS and control rats. There was a significant effect of gender, but not MS, on the total distance traveled (*F*_(1, 71)_ = 9.22, *p* = 0.003). **D** The % time spent in the open arms of the EPM was significantly different between genders (*F*_(1, 71)_ = 22.61, *p* < 0.0001) and there was significant interaction between MS and gender (*F*_(1, 71)_ = 8.10, *p* = 0.006). Pairwise comparison revealed a significant effect of MS in males (control 13.9 ± 1.9 %, MS 6.0 ± 1.0 %, *p = 0.002, Mann–Whitney) but not in females (control 14.3 ± 1.3 %, MS 15.4 ± 1.8 %). **E** The number of entries to the open arms in the EPM was significantly different between genders (*F*_(1, 71)_ = 33.04, *p* < 0.001) and between control and MS groups (*F*_(1, 71)_ = 4.53, *p* < 0.04). Post-hoc comparison indicated a significant effect of MS treatment in males (control 4.2 ± 0.5, MS 2.5 ± 0.4, *p = 0.03, Holm–Sidak) but not in females (control 6.3 ± 0.5, MS 6 ± 0.5).

MS is a widely used animal model of early life adversity. The stress induced by the separation protocol early in life associates with higher anxiety-like behavior in the adult stage, typically observed in the standard behavioral test protocols, open field (OF), and elevated plus maze. Accordingly, the male rats that had experienced MS spent significantly less time in the central zone of the OF arena and in the open arms of the plus maze, as compared to littermate controls (Fig. 1). Interestingly, this behavioral phenotype was not observed in MS females (Fig. 1).

**Fig. 2:**
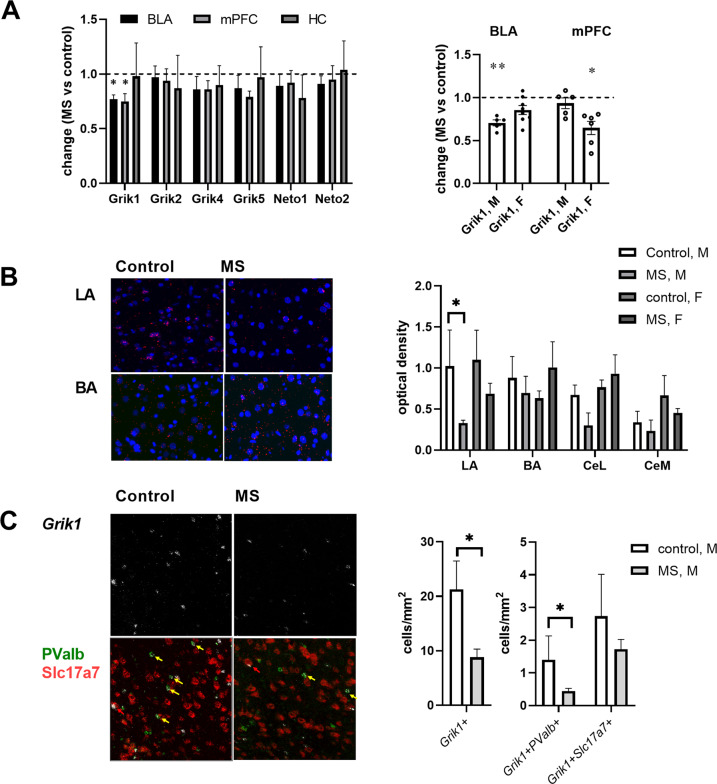
Maternal separation associates with downregulation of *Grik1* expression in non-glutamatergic neurons in the LA in male rats. **A** RT-qPCR analysis of *Grik1-5* and *Neto1-2* gene expression in hippocampus (HC), basolateral amygdala (BLA) and medial prefrontal cortex (mPFC) in MS and control rats. The pooled data are collected from 6–17 samples/group (*n* values for BLA, mPFC and HC: *Grik1* 17,11,6 *Grik2* 17,11,6 *Grik4* 13,11,9 *Grik5* 14,11,9 *Neto1* 15,11,9 *Neto2* 13,11,9) and shows the level of gene expression in MS animals, normalized to controls. BLA: 72 ± 8 %, *p < 0.05, two-tailed *t*-test; mPFC: 75 ± 7 %, *p < 0.05, Mann–Whitney. Gender-specific effects: BLA 69 ± 8 %, *n* = 6 (M) and 76 ± 11 %, *n* = 10 (F), **p < 0.005, two-tailed *t*-test; mPFC 99 ± 6 %, *n* = 5 (M) and 63 ± 10 %, *n* = 6(F), *p < 0.05, Mann–Whitney. **B** ISH illustrating *Grik1* mRNA expression (red) in LA and BA in male control and male MS rat. Parallel DAPI staining of cell nuclei is shown in blue. Pooled data is from three male and three female rats/group. *p < 0.05, two-tailed *t*-test. **C** Triple-ISH staining for *Grik1* (white*)*, *PValb* (parvalbumin, green) and *Slc17a7* (VGlut1, red) in the LA in control and MS male rats. In MS treated rats, the density of all *Grik1* positive *(Grik1*+*)* cells, cells expressing *Grik1* alone as well as *Grik1*+*PValb*+ double-positive cells (yellow arrows) was significantly reduced as compared to controls (*Grik1*+ *p* = 0.002, Mann–Whitney; *Grik1* alone *p* = 0.001, *t*-test; *Grik1*+*PValb*+ *p* = 0.04, Mann–Whitney). The density of Grik1+*Slc17a7*+ (red arrows) was not significantly different between the groups. Pooled data are from three male rats/group.

After validating the model, we went on to investigate whether MS affects KAR expression in various brain areas implicated in anxiety using RT-qPCR. Expression of mRNAs encoding most of the KAR subunits (*Grik2*-*5*) and their NETO auxiliary subunits (*Neto1* and *Neto2*) was not significantly different between adult (P50) control and MS rats in the brain regions studied (hippocampus (HC), basolateral amygdala (BLA), and medial prefrontal cortex (mPFC)). However, *Grik1* mRNA levels in BLA and mPFC were significantly lower in MS group as compared to controls (Fig. 2A). In mPFC, MS-associated downregulation of *Grik1* was significant in females but not in males while in BLA, significant downregulation of *Grik1* was observed only in males (Fig. 2A). Interestingly, in contrast to the adult stage (P50), there were no significant differences in the *Grik1* mRNA levels between control and MS rats in BLA immediately after MS (P14) (MS vs control, 114 ± 3 %, *n* = 7; Males 115 ± 6 %, Females 113 ± 3 %). These data show that MS associates with downregulation of *Grik1* expression in the brain areas implicated in anxiety-like behaviors and that *Grik1* downregulation in male LA parallels the aberrant behavioral phenotype.

The effect of MS on *Grik1* expression in the amygdala nuclei was studied in more detail using in situ hybridization (ISH). In the LA, the mean optical density of the *Grik1* ISH signal was lower in the MS group of animals as compared to controls, being significant in males (Fig. 2B). No significant differences between the groups were detected in other nuclei of the amygdala. To study the cell-type-specific expression pattern in male LA in more detail, we did triple-ISH using probes against *Grik1* together with *Slc17a7* (VGlut1), a marker for glutamatergic neurons, and *Pvalb* (parvalbumin), expressed in fast-spiking GABAergic neurons. In control rats, 12 ± 3.7% of *Grik1*+ cells were co-stained with *Slc17a7*, indicating that GluK1 mRNA is mainly expressed in non-glutamatergic (presumably GABAergic) neurons in the amygdala (Fig. 2C). Interestingly, however, in certain cortical regions, a prominent co-localization of *Slc17a7* and *Grik1* ISH signal was observed (Supplementary Fig. 1), indicating that the cell-type-specific expression pattern of *Grik1* varies between brain regions. A subpopulation (27 ± 4.7 %) of *Pvalb*+ neurons were stained with *Grik1*, and the *Parv*+*Grik1*+ double-positive cells represented 8.5 ± 2.8 % of *Grik1*+ neurons in LA (Fig. 2C). In MS rats, the density of *Grik1*+ cells as well as *Parv*+*Grik1*+ double-positive cells was significantly lower as compared to controls, while no significant differences between groups were detected in the density of *Slc17a7*+*Grik1*+ cells (Fig. 2C). Thus, MS results in strong downregulation of *Grik1*+ expression in non-glutamatergic neurons, including parvalbumin-positive interneurons in the amygdala.

### KAR regulation of glutamatergic transmission in the LA is not significantly affected by MS

Ex vivo electrophysiological recordings from control and MS rats were done to understand how ELS and the associated loss of *Grik1* expression affects the function of the amygdala circuits implicated in anxiety-type behaviors. Previous work has shown that GluK1 subunit containing KARs contribute to glutamatergic synaptic transmission and plasticity in both cortical and thalamic inputs to the principal neurons (PNs) in the LA [8, 9, 17, 25–27]. We found that application of ACET (200–500 nM), a selective antagonist of GluK1 subunit containing KARs [28] resulted in a small but significant depression of the cortical EPSC in the control but not in the MS group (Supplementary Fig. 2A). This effect was significant in female but not male rats and was associated with an increase in paired-pulse ratio (PPR), consistent with a presynaptic mechanism (Supplementary Fig. 2B). In contrast, ACET had no significant effect on EPSCs in thalamic inputs (Supplementary Fig. 2C). The loss of presynaptic GluK1 regulation of transmission at cortico-amygdaloid synapses in MS rats is consistent with observed downregulation of *Grik1* expression in the female cortex (Fig. 2A). However, since the behavioral changes induced by MS were predominant in males, this mechanism is unlikely to contribute to the anxiety-like phenotype.

### GluK1 regulation of GABAergic transmission is perturbed following MS

**Fig. 3:**
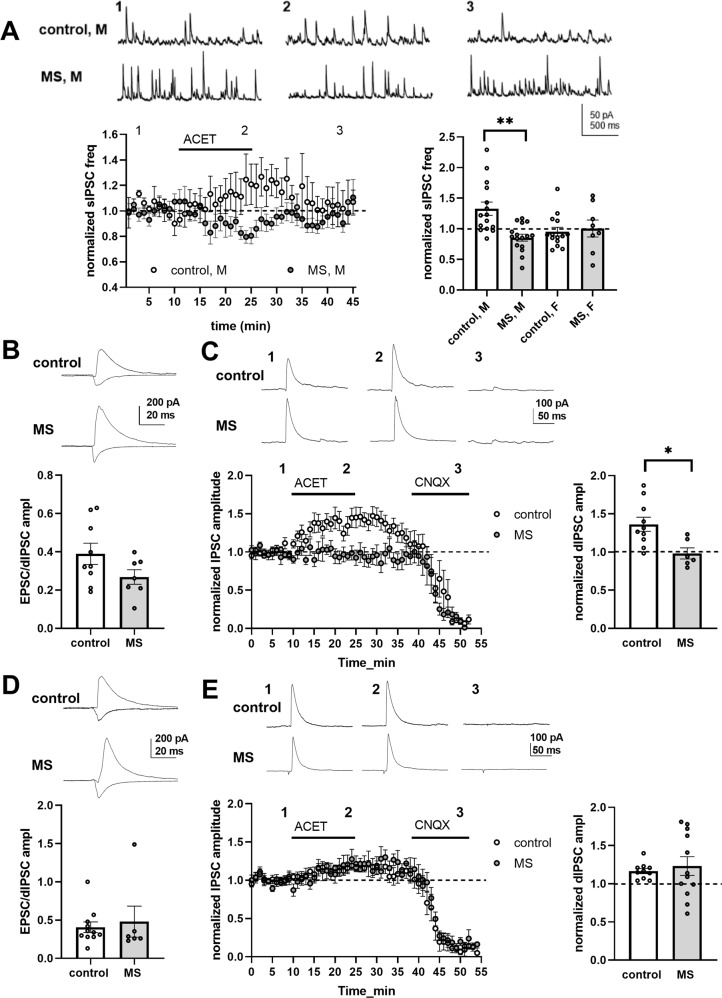
MS is associated with a loss of KAR mediated disinhibition in LA microcircuit responding to activation of cortical but not thalamic inputs. **A**. Example traces from time points illustrated, and a time course plot showing the effect of GluK1 KAR antagonist ACET (200 nM) on the frequency of spontaneous inhibitory postsynaptic currents (sIPSCs) in LA PNs in control and MS male rats (control, M *n* = 15; F *n* = 14; MS, M *n* = 16; F *n* = 8). ACET effect normalized to baseline, in control males, 1.51 ± 0.21, *p* = 0.022 paired *t*-test; MS males, 0.86 ± 0.05, *p* = 0.037, paired *t*-test; difference between groups *p < 0.005, Mann–Whitney. **B** The strength of feedforward inhibition in cortical inputs to LA PNs in slices from control and MS male rats, assessed as EPSC/disynaptic IPSC (dIPSC) ratio (0.38 ± 0.05, *n* = 9 and 0.27 ± 0.04, *n* = 7, respectively). Example traces and analyzed data from individual cells. *t*-test between groups, *p* = 0.10. **C** The effect of ACET on cortical dIPSCs in LA PNs in control and MS male rats. Example traces from baseline (1), during ACET application (2) and after drug washout (3), and a time course plot of normalized dIPSC amplitude. CNQX was applied in the end of the experiment to assure disynaptic response. Effect of ACET on the dIPSC amplitude in individual experiments, normalized to the baseline level (control 1.36 ± 0.09, *n* = 10, *p* = 0.004, paired *t*-test; MS 0.98 ± 0.07, *n* = 6, *p* = 0.95, paired *t*-test). Difference between groups *p < 0.05. **D** Similar data as in (**B**) for EPSC/dIPSC ratio in thalamic inputs to LA PNs in control and MS male rats (control 0.41 ± 0.07, *n* = 11; 0.48 ± 0.19, MS *n* = 6), *t*-test between groups, *p* = 0.67. **E** The effect of ACET on thalamic dIPSCs in LA PNs in control (*n* = 10) and MS (*n* = 11) male rats. The data shown as in (**C**). ACET effect in control group, 1.17 ± 0.03, *p* = 0.03, paired *t*-test; MS group 1.23 ± 0.12, *p* = 0.51, paired *t*-test; difference between groups, *p* = 0.84 Mann–Whitney test. All the statistics were calculated using raw (non-normalized) data.

GluK1 is expressed in various types of GABAergic interneurons and is involved in regulation of their excitability as well as GABA release ([29]; reviewed by Lerma [7]). After MS, downregulation of GluK1 mRNA was predominant in non-glutamatergic neurons, presumably representing GABAergic interneurons in the LA (Fig. 2C). To investigate whether GABAergic transmission was affected in rats that had experienced MS, we studied the effect of ACET on sIPSCs (spontaneous inhibitory postsynaptic currents) in LA. ACET application caused a significant increase in the sIPSC frequency in LA PN’s in control males, while a small decrease in sIPSC frequency was observed in the MS males (Fig. 3A). ACET had no effect on sIPSCs in the females in either group (Fig. 3A). Basal sIPSC frequency was not significantly different between the groups, when comparing data from all animals (control 15.2 ± 1.4 Hz, MS 15.7 ± 1.2 Hz; *p* = 0.78, *t*-test) and males and females separately (Males, control 13.4 ± 1.7 Hz, MS 16.4 ± 1.2 Hz, *p* = 0.197, *t*-test; Females, control 17.5 ± 2.1 Hz, MS 14.7 ± 2.5 Hz, *p* = 0.43; *t*-test). No differences in sIPSC amplitudes between groups or as a result of ACET application were detected (Supplementary Table 1).

To control whether the effect of ACET on sIPSCs was mediated via a change in the network excitability or GABA release, we tested the effect of ACET on the frequency of action potential independent (miniature) IPSCs (mIPSCs) in control male rats. In a few individual cells, ACET application resulted in an increase (one out of six cells) or a decrease (two out of six cells) in mIPSC frequency (Supplementary Fig. 3A). However, when pooling data from all recorded cells, there was no overall effect of ACET on either frequency or amplitude of mIPSCs (Supplementary Fig. 3A; Supplementary Table 1). Furthermore, separating the events into fast (rise time 0–2 ms) and slow (rise time >3 ms) subpopulations, expected to represent events from proximal or distal GABAergic synapses, did not reveal significant effects of ACET on mIPSC frequency (Supplementary Fig. 3A). Since KARs might selectively regulate synchronous GABA release [30, 31], we also studied the effect of ACET on monosynaptic IPSCs, evoked by stimulating GABAergic interneurons close to the recorded neuron in male controls. CNQX was applied at the end of the experiment to ensure the monosynaptic nature of the response. ACET application significantly increased IPSC amplitude in three out of eight cells and in one cell, a decrease in IPSC amplitude was observed (Supplementary Fig. 3B). Again, however, there was no significant effect of ACET on IPSCs or on PPR of IPSCs when all the data were pooled (Supplementary Figure 3B). These data indicate that GluK1-dependent regulation of GABAergic transmission mainly depends on changes in circuit excitability rather than GABA release.

To understand the role of GluK1 receptors in the LA microcircuit in more detail, we investigated feedforward inhibition in both, cortical and thalamic inputs to LA PN’s. Based on the previous results, only males were included in these experiments. Monosynaptic EPSC was first recorded at −70 mV, after which the cell was clamped 0 mV isolate disynaptic IPSC (dIPSC). From these data we analyzed two parameters: (1) the strength of feedforward inhibition by measuring EPSC/dIPSC ratio in control and MS group of animals and (2) the effect of KAR antagonism on feedforward inhibition, by studying the effect of ACET on dIPSC amplitude. Again, CNQX was applied at the end of the experiment to confirm the disynaptic nature of the response.

At cortical inputs, the strength of feedforward inhibition was slightly, but not significantly (*p* = 0.10, unpaired *t*-test), lower in the MS group as compared to controls (Fig. 3B) while there was no difference between the groups in the thalamic inputs (Fig. 3D). Blocking GluK1 KARs with ACET resulted in a significant increase in the amplitude of disynaptic IPSC in cortical inputs and this effect was completely lost in the MS group (Fig. 3C). At thalamic inputs, a small increase in the dIPSC amplitude in response to ACET application was observed in slices from both, control and MS rats, and there were no differences in the effect of ACET between the groups (Fig. 3E).

These data indicate that endogenous KAR activity attenuates feedforward inhibition in LA, i.e., disinhibits PNs during activation of both cortical and thalamic afferents. This KAR-mediated disinhibition is downregulated after MS in a pathway-specific manner, in cortical but not in thalamic inputs to the LA.

### GluK1 subunit-containing KARs are endogenously activated in the parvalbumin-positive GABAergic interneurons to control their excitability

GABAergic inhibition in the BLA is mediated via a complex microcircuit of various types of inhibitory interneurons, which all have their specific functions in the circuit. mRNAs encoding KAR subunits are expressed non-selectively in different types of GABAergic interneurons [29, 32]; however, their neuron subtype-specific functions are poorly characterized.

**Fig. 4:**
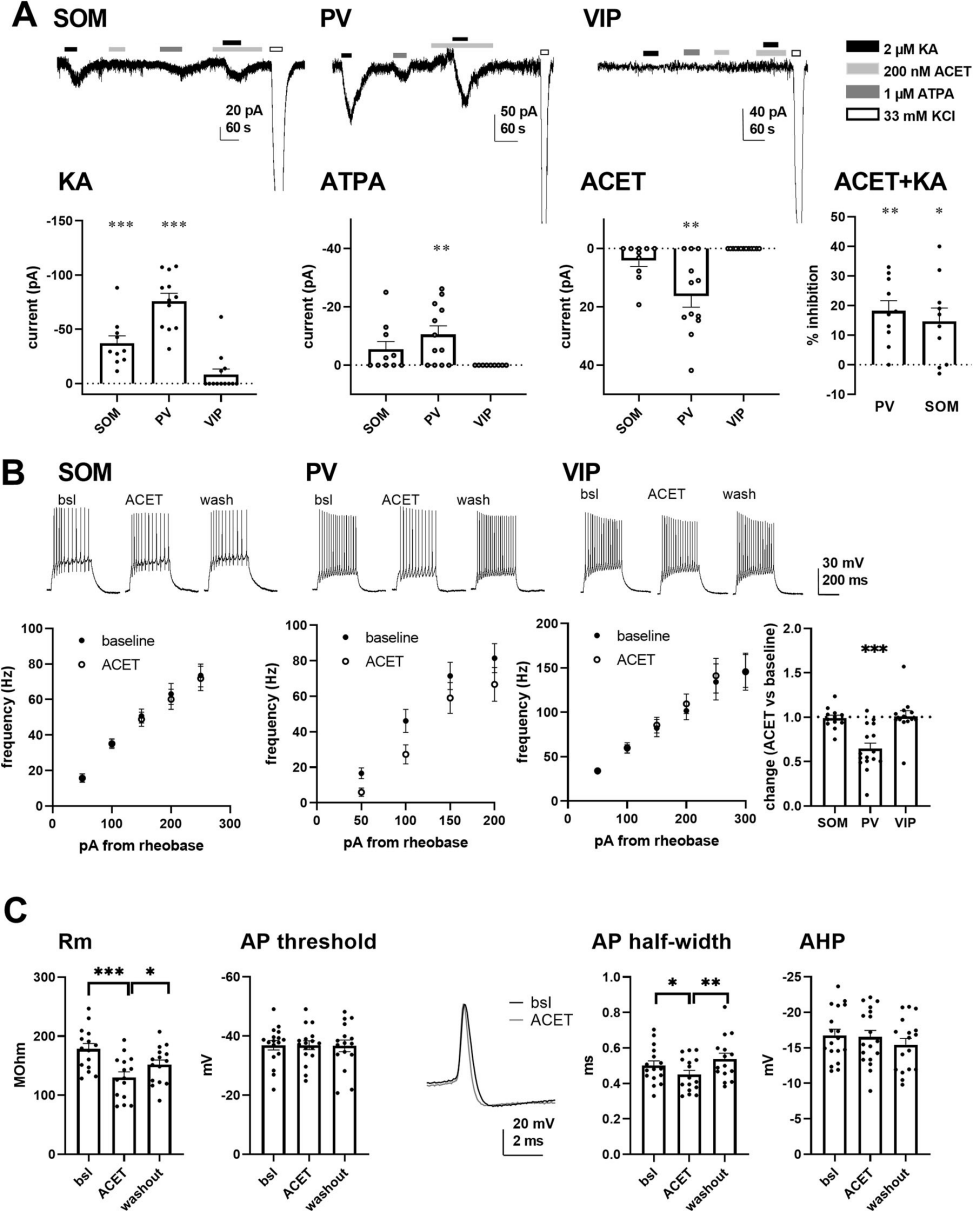
Characterization of KAR-mediated ionotropic currents and effect of GluK1 antagonism on intrinsic excitability in PV+, SOM+, and VIP+ interneurons in the LA. **A** Pharmacological characterization of ionotropic KARs in PV+ SOM+, and VIP+ neurons in the LA. Example traces and analyzed data on the amplitude of KA (2 µM), ATPA (1 µM) and ACET (200 nM) induced currents in different types of GABAergic neurons in the LA (*n* = 10,12, and 13, for SOM, PV and VIP, respectively). ***p < 0.001, **p < 0.005, single group *t*-test. % inhibition of the KA current by ACET (200 nM) for PV (18 ± 3.3%, *p* < 0.005, paired *t*-test) and SOM (15 ± 4.3 %, *p* < 0.05, paired *t*-test) neurons. **B** Effect of ACET on the intrinsic excitability of PV+, SOM+, and VIP+ neurons in the LA. Example traces and pooled data on the firing frequency in response to depolarizing steps (50–300 pA). The bar graph shows the effect of ACET on firing frequency in response to 100 pA depolarizing step in different cell types (frequency in ACET, normalized to control in SOM+, 0.99 ± 0.03, *n* = 12; PV+, 0.65 ± 0.06, *n* = 16; VIP+, 1.01 ± 0.06, *n* = 13; ***p < 0.001, paired *t*-test). **C** Effect of ACET on input resistance, AP threshold, AP half-width and AHP amplitude in LA PV cells (*n* = 16). ***p < 0.001, *p < 0.05, paired *t*-test.

To investigate the effect of KARs on the excitability of specific interneuron subtypes in the BLA, mice expressing Cre recombinase in parvalbumin (PV), somatostatin (SOM), and vasoactive intestinal polypeptide (VIP) expressing cells were crossed with *Cre*-reporter mice (Cre-dependent TdTomato), which allowed visualization and recording of the specific interneuron subpopulations. We first used pharmacological approach to check whether functional ionotropic KARs could be detected in the various cell types. Application of kainate (2 µM) in the presence of selective antagonists for AMPA, NMDA, and GABA receptors (2 µM NBQX, 50 µM APV and 100 µM PiTX, respectively), expected to selectively activate KARs in all different subunit combinations, produced a significant inward current in SOM+ and PV+ neurons but not in VIP+ interneurons in adult mouse LA (Fig. 4A). GluK1 specific agonist ATPA (1 µM) produced a small but significant inward current only in PV+ cells, while GluK1 antagonist ACET partially inhibited KA-induced inward current both in SOM+ and PV+ neurons (Fig. 4A). Interestingly, ACET application alone resulted in a detectable outward current in most PV+ neurons (9 out of 12 cells). No significant differences in the current amplitudes were observed between males and females for any of the drugs. Thus, SOM+ and PV+ interneurons express somatodendritic KARs that can enhance their excitability via ionotropic depolarization and these receptors are endogenously activated in PV+ cells.

Since GluK1 KAR may also regulate neuronal excitability via G-protein coupled mechanisms [33], we also tested the effect of ACET on intrinsic excitability of the different cell types. Interestingly, while having no effect on the excitability of SOM+ and VIP+ cells, ACET application robustly and reversibly attenuated action potential (AP) firing in most of (9 out of the 16 cells) the recorded PV+ interneurons in response to a depolarizing current pulse (Fig. 4B). The effect of ACET on the firing rate of PV neurons did not correlate with changes in Vm (Pearson’s correlation, *r* = 0.146, *p* = 0.576) and a significant reduction in the firing rate was observed also in cells where ACET application had no effect (<1 mV) on Vm (frequency in ACET 57 ± 5% of control, *n* = 6, *p* = 0.007, paired *t*-test). ACET had no effect on AP threshold or on the amplitude of the afterhyperpolarizing potential (AHP), measured after the 3rd spike in the train. However, both the input resistance and spike half width were significantly and reversibly reduced in response to ACET application (Fig. 4C).

These results indicate that while GluK1 KARs are expressed in various types of GABAergic interneurons, they are endogenously activated specifically in the PV+ neurons to control their excitability.

### GluK1 activity indirectly attenuates recruitment of somatostatin positive interneurons in the feedforward inhibitory circuit

In BLA, PV+ neurons contribute to feedforward inhibition via direct projections to the soma of the PNs, but also indirectly, via SOM+ neurons [34]. The indirect mechanism disinhibits PNs and might thus explain the actions of endogenous KARs in the LA microcircuit. Current-clamp recordings of stimulus-induced EPSPs were made from PV+, SOM+, and VIP+ interneurons to investigate the contribution of GluK1 KARs to interneuron firing in response to cortical input. The stimulation intensity was adjusted to the AP threshold so that only some of the evoked EPSP triggered AP under basal conditions.

**Fig. 5:**
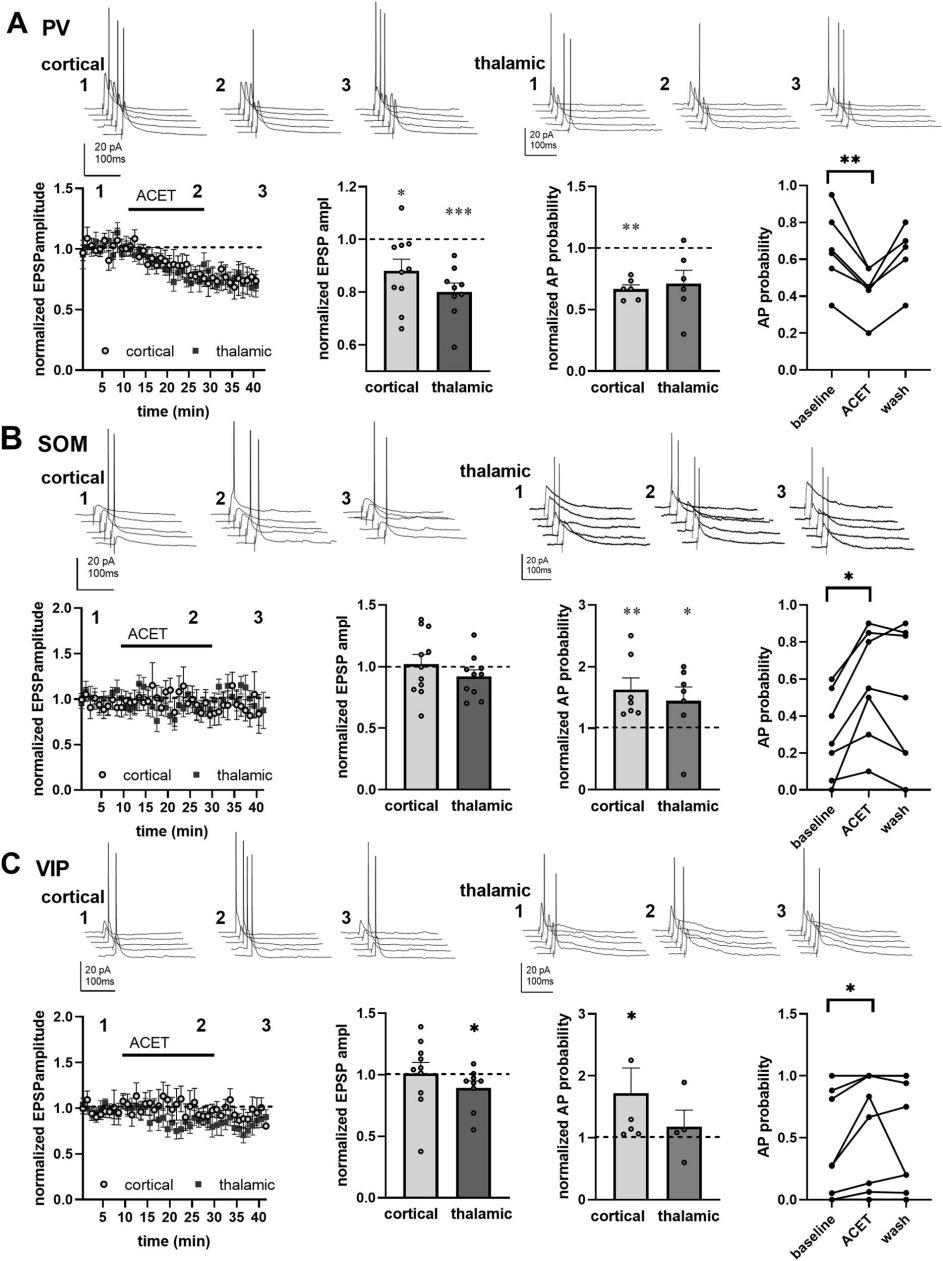
Endogenous activity of GluK1 KARs differentially regulates recruitment of various types of GABAergic interneurons in the feedforward inhibitory circuit. A Effect of ACET on EPSPs and AP firing probability in LA PV+ neurons. Example EPSP triggering AP firing in about 50% of trials before application of ACET, evoked by stimulation of cortical and thalamic afferents. Pooled data on the effect of ACET on EPSP amplitude (0.88 ± 0.04, *n* = 10 and 0.80 ± 0.03, *n* = 9, for cortical and thalamic EPSPs, respectively) and AP firing probability (cortical, 0.67 ± 0.03, *n* = 6; thalamic 0.71 ± 0.10, *n* = 6). Data with raw values for AP firing probability before, during and after ACET application is shown for cortical inputs. ***p < 0.001; **p < 0.01, *p < 0.05; paired *t*-test. **B** Similar data for SOM + neurons in LA. Normalized EPSP amplitude, 1.02 ± 0.07, *n* = 11 and 0.92 ± 0.05, *n* = 10; normalized AP probability, 1.63 ± 0.17, *n* = 7 and 1.45 ± 0.21, *n* = 7 for cortical and thalamic inputs, respectively. **p < 0.01, *p < 0.05; paired *t*-test. **C** Similar data for VIP+ neurons in LA. Normalized EPSP amplitude, 1.01 ± 0.09, *n* = 10 and 0.89 ± 0.05, *n* = 9; normalized AP probability, 1.72 ± 0.37, *n* = 6 and 1.18 ± 0.23, *n* = 4 for cortical and thalamic inputs, respectively. *p < 0.05; paired *t*-test.

In PV+ neurons, application of ACET resulted in significant reduction in the EPSP amplitude in both cortical and thalamic inputs and reduced the probability of AP firing in response to activation of cortical inputs (Fig. 5A). In SOM+ neurons, ACET application had no effect on the EPSP amplitude, but significantly increased the probability of AP firing to afferent activation (Fig. 5B). In VIP+ neurons, ACET application resulted in small depression of EPSP amplitude in thalamic inputs but had no effect on cortical inputs and significantly increased AP firing in response to activation of cortical inputs in a subset of the cells (Fig. 5C).

**Fig. 6:**
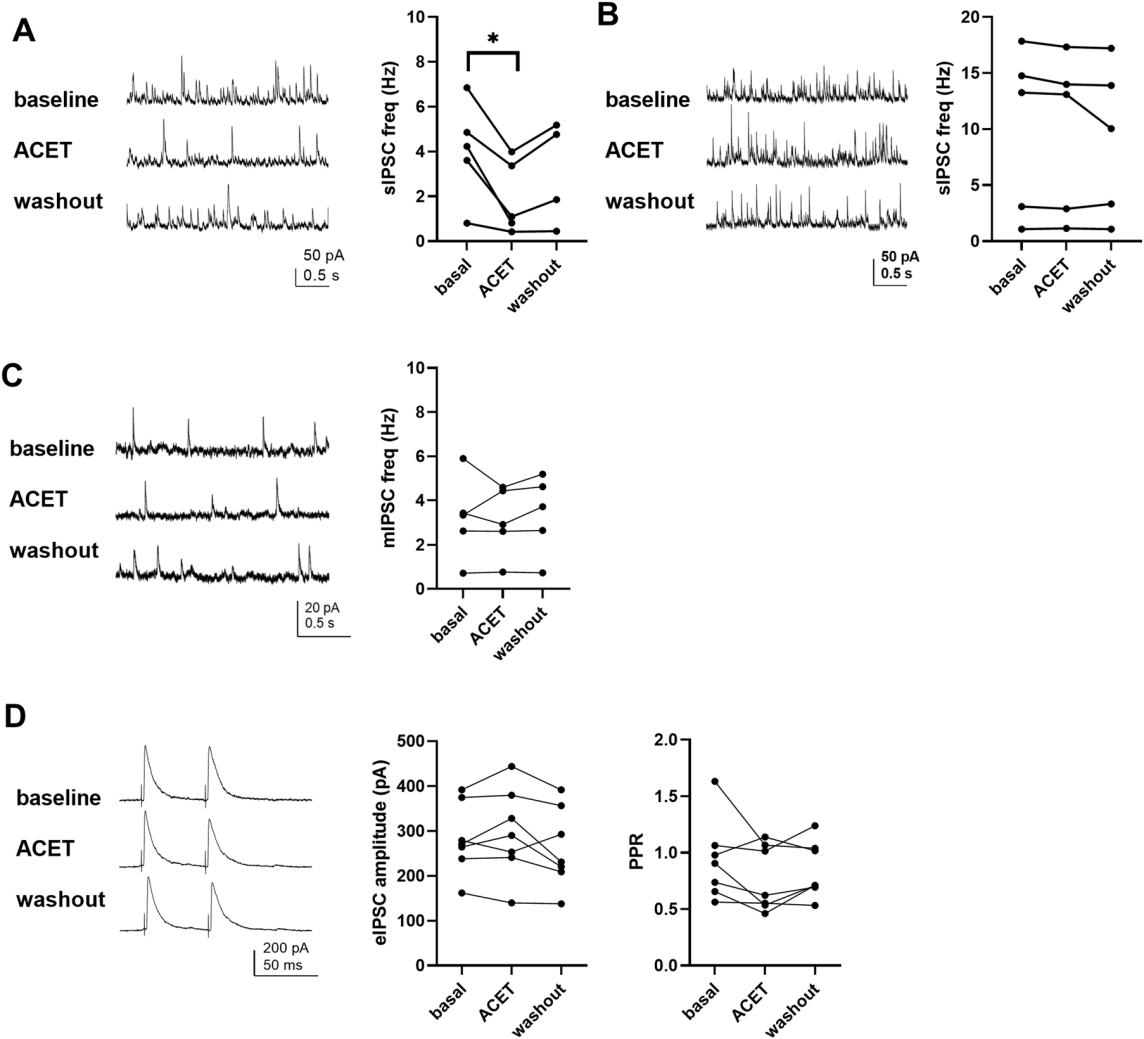
Endogenous activity of GluK1 KARs regulates GABAergic input to SOM+ neurons. **A** Effect of ACET on sIPSC frequency in SOM+ neurons in LA. Example traces and analyzed data for mean sIPSC frequency in baseline (4.1 ± 0.88 Hz), after ACET application (1.9 ± 0.65 Hz) and after drug washout (3.1 ± 0.89 Hz) in individual neurons. *n* = 5, *p < 0.05, paired *t*-test. **B** Similar data on sIPSC frequency in PV+ neurons (*n* = 5). sIPSC frequency in baseline 10.0 ± 3.3 Hz, ACET 9.7 ± 2.9 Hz and after drug washout 9.1 ± 2.7 Hz. **C** Effect of ACET on mIPSC frequency in SOM+ neurons in LA (*n* = 5). Data shown as in (**C**). mIPSC frequency in baseline 3.2 ± 0.74 Hz, ACET 3.1 ± 0.70 Hz and after drug washout 3.4 ± 0.80 Hz. **D** Effect of ACET on amplitude and PPR of evoked IPSC (eIPSCs) in LA SOM + neurons (*n* = 7).

SOM+ neuron firing in the BLA is strictly controlled by GABAergic input, originating from PV+ and VIP+ interneurons [34]. Recording of GABAergic synaptic events revealed that ACET application resulted in reversible depression of sIPSC frequency in SOM+ neurons (Fig. 6A) but had no effect on sIPSCs in PV+ neurons (Fig. 6B). sIPSC amplitude was not affected in either cell type (Supplementary Table 1). ACET had no significant effect on mIPSCs or on PPR of evoked IPSCs in SOM+ neurons (Fig. 6C, D), indicating that the loss of sIPSCs in response to KAR antagonism depended on the excitability of the afferent GABAergic neurons.

These data are consistent with a hypothesis that GluK1 regulates the excitability of PV+ neurons, which gate AP firing in SOM+ neurons via inhibitory GABAergic connections. This shift in the excitability of SOM+ and PV+ interneurons can fully explain the KAR-dependent disinhibition of the principal cells during afferent activation. Yet, additional modulation via VIP+ neurons cannot be excluded.

### Local inactivation of GluK1 in the amygdala leads to a mild anxiety-like phenotype

**Fig. 7:**
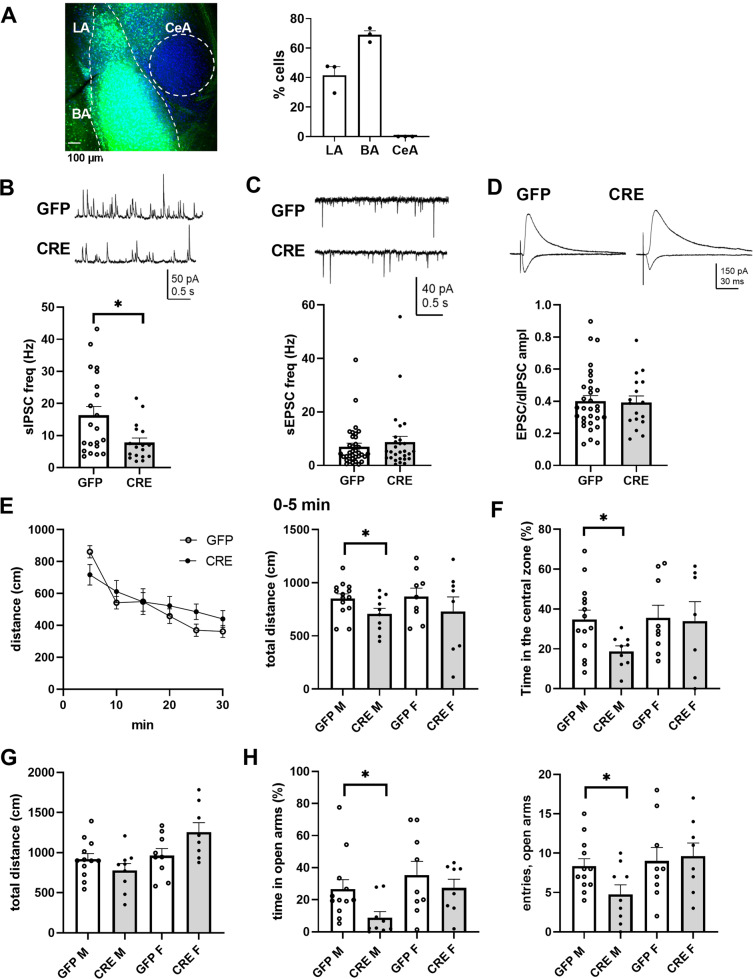
Local inactivation of GluK1 expression in the BLA reduces GABAergic transmission in LA and increases anxiety-like behavior in male mice. **A** GFP fluorescence in the BLA of *Grik1*^*tm1c*^ mice 3 weeks after injection of GFP-CRE AAV virus. % of GFP expressing neurons of DAPI stained cells in LA and BA (*n* = 3). **B** Frequency of sIPSCs in LA principal neurons of male *Grik1*^*tm1c*^ mice 3–4 weeks after injection of AAV virus encoding for CRE-EGFP (CRE) or GFP only. The mean frequency of sIPSCs was significantly lower in neurons in CRE injected mice (7.8 ± 1.4 Hz, *n* = 17) as compared to GFP controls (16.3 ± 2.7 Hz, *n* = 19; *p* = 0.016, Mann–Whitney). The mean amplitude of sIPSCs was not different between the groups (GFP 21.0 ± 1.1 pA, CRE 23.7 ± 1.4 pA). **C** Frequency of sEPSC from the same cells as in (**B**). GFP 7.0 ± 1.1 Hz, CRE 8.7 ± 2.1 Hz; sEPSC amplitude GFP 14.1 ± 0.5 pA CRE 14.7 ± 1.0 pA. **D** The strength of feedforward inhibition in cortical inputs to LA PNs in slices from GFP and CRE injected male *Grik1*^*tm1c*^ mice, assessed as EPSC/dIPSC ratio (0.40 ± 0.03, *n* = 31 and 0.39 ± 0.04, *n* = 17, respectively). Example traces and analyzed data from individual cells. **E** Total distance traveled in the open field arena during the 30 min of testing for all animals (CRE *n* = 18, GFP n = 23), and during the first 5 min for males and females separately (GFP M, 853 ± 41 cm, *n* = 14, CRE M, 706 ± 50 cm, *n* = 10, GFP F, 872 ± 73 cm, *n* = 9, CRE F, 730 ± 127 cm, *n* = 8). **F** % time spent in the central zone during the first 5 min of testing in the open field (GFP M, 35 ± 4.6 %, CRE M, 19 ± 2.5 %, GFP F, 36 ± 5.9 %, CRE F, 34 ± 9.0 %). No differences between the groups were detected with two-way ANOVA, while a significant difference between GFP and CRE injected males, but not females, was revealed with pairwise comparison; *p < 0.05, two-tailed *t*-test. **G** Total distance traveled (5 min of testing) in an elevated plus maze test. Data are shown separately for male and female animals in CRE or GFP injected groups. The total distance was affected by gender (*F*_(1, 34)_ = 8.59, *p* = 0.006), but not treatment. **H** % time and number of entries to the open arms. The % time spent in open arms was significantly different between CRE and GFP injected groups (*F*_(1, 34)_ = 4.25, *p* = 0.04) and between genders (*F*_(1, 34)_ = 4.75, *p* = 0.03; GFP M, 27 ± 5.7 %, *n* = 12; CRE M, 8.9 ± 3.6 %, *n* = 9; GFP F, 35 ± 8 %, *n* = 9; CRE F, 27 ± 5.1 % *n* = 8). Pairwise comparison indicated a significant effect of MS in males (p = 0.02, Mann–Whitney), but not in females. Two-way ANOVA detected no significant differences in entries to open arms, while pairwise comparison indicated a significant difference between GFP and CRE injected males but not females (GFP M, 8.3 ± 0.9; CRE M, 4.7 ± 1.1; GFP F, 9.0 ± 1.6; CRE F, 9.6 ± 1.6; * p < 0.03, two-tailed *t*-test).

Pharmacological and genetic evidence suggests that GluK1 subunit-containing KARs regulate anxiety-like behaviors in rodents [11, 15, 17]; however, whether these effects depend on *Grik1* expression in the amygdala remains unknown. To confirm this, we used a conditional knockout mouse model allowing spatially and temporally restricted inactivation of *Grik1* in the adult amygdala using local injection of EGFP-CRE encoding AAV viruses [13]. Electrophysiological analysis and behavioral testing of these mice as well as littermate controls injected with EGFP encoding AAV viruses was carried out 3–4 weeks later. Post-hoc morphological characterization was used to confirm the site of injection and estimate the infection rate (average of 55 ± 15 % of DAPI positive cells in BLA; Fig. 7A).

GluK1 knockdown in the BLA resulted in significant reduction in GABAergic transmission, observed as lower sIPSC frequency in LA PN’s in slices from CRE injected male mice as compared to controls (Fig. 7B). No difference in glutamatergic transmission (sEPSC frequency and amplitude, Fig. 7C) or in the strength of feedforward inhibition (EPSC/dIPSC ratio) in response to activation of cortical afferents was detected (Fig. 7D). Thus, chronic GluK1 inactivation resulted in changes in ongoing GABAergic transmission in LA, yet these effects were distinct from those induced by acute pharmacological inhibition of GluK1 function. These data indicate that GluK1 expression has an additional role in maintaining GABAergic transmission in the LA.

Behavioral testing of the mice in the OF arena revealed no differences between the groups in the total distance traveled during 30 min of testing (Fig. 7E). However, during the first 5 min, CRE injected male mice traveled shorter distance as compared to the GFP injected controls and avoided the central zone (Fig. 7E, F), while no differences between the groups were detected in females. Similarly, in the elevated plus maze, CRE injected male but not female mice spent significantly less time in the open arms and made less open-arm entries as compared to controls (Fig. 7H). Again, there was no difference in the total distance traveled (Fig. 7G). These results indicate that the loss of *Grik1* expression in ~50% of the cells in BLA is sufficient to reduce GABAergic tone in the LA and increase anxiety-like behavior in male mice.

## Discussion

Amygdala GABAergic circuitry has been critically implicated in regulation of fear and anxiety, including stress-induced pathological anxiogenesis [35–38]. The overall scenario is that GABAergic local interneurons constrain the activation of anxiety-promoting projection neurons in the amygdala, and the loss of this inhibitory control results in excessive response to anxiogenic stimuli. However, it has become apparent that the BLA comprise intermingled populations of neurons contributing to distinct effector networks, which react differentially in anxiety-like states [39, 40]. Hence, anxiogenic development likely involves multifaceted functional reorganization of the GABAergic microcircuits, which may regulate excitability of PNs in a cell-type- and projection-specific manner. Here, we identify a novel KAR-dependent mechanism that modulates the LA GABAergic microcircuit in a cell-type-specific manner and show that this mechanism is downregulated in male rats displaying increased anxiety-like behavior after early life stress.

KARs and in particular, GluK1 subunit-containing KARs, are reported to have various effects on the physiology of GABAergic interneurons [41, 42], but their cell-type-specific functions are not well characterized. In the BLA, PV, and SOM expressing GABAergic neurons mediate feedforward inhibition of the PNs, and predominantly target perisomatic region and distal dendrites of the PNs, respectively [43]. The VIP expressing interneuron population mainly targets other GABAergic interneurons, providing a potent mechanism gating their excitability [44, 45]. We detected KAR-mediated inward currents that were partially blocked by GluK1-specific antagonist in PV+ and SOM+ neurons, but interestingly, not in VIP+ neurons despite these cells’ strong expression of mRNA encoding GluK1 and GluK2 KAR subunits [32]. Direct depolarization of SOM+ and PV+ neurons can explain the robust recruitment of GABAergic activity in response to KAR activation, observed in various regions of the brain including BLA [10, 11]. Endogenously active KARs were detected in the PV+ neurons, where application of GluK1 selective antagonist ACET resulted in significant reversible decrease in their intrinsic excitability.

In addition to their ionotropic activity, GluK1 KARs may activate G-protein coupled signaling pathways to tonically inhibit potassium channels and consequently, increase neuronal excitability (reviewed by Valbuena and Lerma [33]). The activity of voltage-gated potassium channels has a strong impact on PV cell excitability and can be modulated via second messengers [46], thus providing potential molecular targets for KAR modulation in PV neurons. However, the exact molecular mechanism by which endogenous activity of KARs regulates excitability of PV neurons remains a topic for further studies.

Since PV neurons densely innervate perisomatic region of the PN [46, 47], a decrease in the excitability of PV neurons in response to KAR antagonism would be expected to weaken GABAergic input to PNs. However, in naive control animals, ACET application was associated with a decrease in PV+ neuron excitability and an increase in sIPSC frequency in LA PNs in vitro. In addition to directly inhibiting PNs, activity of PV+ neurons can dynamically control recruitment of the SOM+ neurons in the feedforward GABAergic circuitry and this indirect PV-SOM-disinhibitory pathway may override direct PV-mediated inhibition of PNs during processing of auditory information [34]. In support to the involvement of SOM+ interneurons in the KAR-mediated disinhibition, KAR antagonism resulted in robust reduction of sIPSC frequency in the SOM+ interneurons in the LA. These data are consistent with a scenario where reduced excitability of PV+ neurons upon KAR antagonism releases inhibitory control of the SOM+ neurons and facilitates their firing in response to afferent activity. Accordingly, increase in SOM+ neuron firing in response to afferent activation was readily observed after KAR antagonism. This indirect PV-SOM-dependent mechanism can fully explain the increase in feedforward inhibition of the LA principal neurons in response to KAR antagonism. Yet, we cannot exclude the possibility that the subpopulation of VIP+ interneurons that increased their firing in response to afferent activation upon KAR antagonism also contribute to this regulation. Enhanced activity of VIP+ neurons may dampen the excitability PV neurons and thereby disinhibit PN’s. In addition, a subpopulation of VIP+ neurons expressing also CCK+CB1+ directly connect to principal neurons [44, 45] and may thereby contribute to the observed increase in GABAergic drive.

Changes in the excitability of LA GABAergic neurons and in particular, PV+ and SOM+ neurons have been previously associated with anxiogenic stressors (e.g. [48–51]; reviewed by Babaev et al. [37]). Early life stress, in particular, affects maturation of the PV+ interneurons in the BLA [52–55], which may result in lasting changes in their function. The level of PV+ neurons activity in the BLA controls anxiety-like behaviors [56]. Regulation of PV neuron excitability via endogenously active KARs has not been shown before, and thus represents a completely new mechanism modulating physiology of the PV neurons in a behaviorally relevant circuitry.

SOM+ interneurons are also a critical part of the amygdala anxiety circuit, and changes in their excitability influence anxiety-like behaviors (reviewed by Prager et al. [35], Babaev et al. [37], Robinson and Thiele [57]). Dendritic inhibition via SOM+ interneurons efficiently gate generation of dendritic Ca+ signals and plasticity induction [58–60]. Therefore, the increase in SOM-mediated dendritic inhibition as an indirect consequence of lost GluK1 KAR function could contribute to the altered threshold for plasticity in LA, observed after early life stress [1].

Hence, the cell-type-specific effects of KARs on GABAergic interneurons may modulate both the excitability and plasticity of the LA microcircuits with prospective behavioral consequences. Although previous work with KAR subunit knockout or overexpressing mice clearly indicate a role for KARs in anxiety-like behaviors [11, 14, 16, 61], it has remained unclear whether the aberrant behaviors can be specifically attributed to KARs located in the amygdala or involve other brain regions [62]. Furthermore, KAR expression has profound effects on the development of neuronal networks (e.g. [13, 63]), which likely contribute to the behavioral phenotype in these mouse lines. Our current data using a conditional knockout mouse indicate that inactivation of *Grik1* in approximately 50% of cells in the adult BLA is sufficient to increase anxiety-like behavior. The changes in *Grik1* mRNA expression after the genetic manipulation are not identical to those observed after MS treatment in terms of the cell-type specificity (i.e. non-specific vs. mainly non-glutamatergic neurons) and the magnitude of decrease (40% vs. 70% of control after genetic inactivation and MS, respectively; [13]). Accordingly, there were differences in the electrophysiological phenotype in the LA. Genetic inactivation of *Grik1*, but not MS, resulted in significant downregulation of sIPSC frequency in the LA, revealing a role for GluK1 expression in maintaining GABAergic tone. Interestingly, the strength of feedforward inhibition that was upregulated in response to acute inhibition of GluK1 remained unaltered. One possibility is that the overall downregulation of GABAergic transmission after *Grik1* knockdown masks the upregulation of disynaptic IPSCs, resulting in no net change in the strength of feedforward inhibition. Alternatively, the disinhibitory effect, predominant during acute inhibition of GluK1 and dependent on changes in PV excitability, might be a target for homeostatic adaptation. PV cells adapt to chronic decreases in activity by facilitating their firing to regain a ‘set point’ using various mechanisms [64–66], which might compensate for the absence of GluK1.

Nevertheless, considering the above differences, both MS and local *Grik1* inactivation resulted in changes in GABAergic transmission in the LA and enhanced anxiety-like behavior. Even though the behavioral phenotype in individual tests was not highly significant, the mice with local loss of *Grik1* in the amygdala displayed a mild anxiety-like phenotype consistently through all the different tests. Interestingly, the behavioral phenotype was only detected in male mice, thus mimicking the gender specificity of anxiety-like behavior after MS in male but not female rats [67]. However, since the estrous cycle affects amygdala function in females [68] and we did not control for this parameter in our experiments, we cannot exclude the possibility some effects of MS in female rats were masked by the influence of estrous cycle.

In summary, we have characterized mechanisms by which GluK1 KARs modulate excitability of the LA GABAergic microcircuit and present evidence suggesting that the loss of this modulation contribute to anxiogenesis after early life stress. Specifically, we show that endogenous activity of GluK1 KARs in the PV interneurons regulates feedforward inhibition of the LA principal neurons during activation of cortical afferents. In addition, we show that GluK1 expression is required for the maintenance of GABAergic transmission in the LA. We propose that the KAR-dependent shift in the excitability of the LA microcircuit perturbs plasticity induction and/or controlled activation of anxiety-promoting projection neurons and thereby contribute to aberrant behaviors after early life stress. Furthermore, our data identifies GluK1 as an interesting target for therapeutic applications against stress-induced anxiety disorders.

## Materials and methods

### Animals

Male and female Wistar rats were used in most experiments. In addition, experiments were performed using the following mouse lines: *Grik1*^*tm1c*/*tm1c*^ [13] and PV-Cre (JAX 008069), SST–Ires-Cre (JAX 028864; kindly provided by Prof Esa Korpi and Anni-Maija Linden) and VIP-IRES-Cre (JAX 031628), crossed with Ai14 tdTomato reporter (JAX 007914). All mice were homozygous and in the C57BL/6J background. All the experiments were done in accordance with the University of Helsinki Animal Welfare Guidelines and approved by the Animal Experiment Board in Finland.

### Maternal separation

MS experiments were performed using Wistar rats. The protocol consisted of daily 180 min separation of the pups and the dam from postnatal day 2 until day 14 (MS group), while littermate controls remained with the dam in their home cage. Pups were assigned to experimental groups randomly. During the separation, the pups were transferred to a separate cage containing individual compartments with bedding. Heat pads were used to maintain body temperature. On postnatal day 21, the litters were weaned and the animals were maintained under standard housing conditions (2–5 animals of the same sex/cage) until adulthood. The animals were sacrificed for experiments between 9 a.m. and 3 p.m., during the light period (lights on 6 a.m., off 6 p.m.). The stage of the estrous cycle was not controlled.

### RT-qPCR and in situ hybridization

BLA, mPFC, and HC were dissected from 400-µm-thick vibratome sections cut from the brain of control and MS Wistar rats at postnatal day (P)50. Purification of total RNA, cDNA synthesis, and the real-time quantitative PCR was carried out essentially as described [13, 63]. All samples were analyzed in triplicate. Relative quantification of gene expression between groups was analyzed using the standard 2-ddCt method.

ISH was carried out on 5-µm-thick paraffin sections from control and MS rat brain using digoxigenin (DIG)-labeled antisense RNA probes against *Grik1* as described [13, 69]. TSA-Plus Cyanine3/Fluorescein System (Perkin Elmer) was used to visualize ISH signal, followed with a standard DAPI staining. Stained sections were imaged using Zeiss Axioimager M2 microscope with Axiocam HRc camera. The level of mRNA expression in the various amygdala nuclei was semiquantitatively determined by measuring the optical density of the ISH signal (red) within anatomically defined area, which was normalized against the quantity of cells (DAPI staining) using ImageJ software. At least three sections were analyzed for each animal, and at least three animals were included in each group.

Triple-ISH was done using the RNA scope^®^ Fluorescent Multiplex kit (Advanced Cell Diagnostics) on fresh frozen brain sections from control and MS rats. The brain was removed under anesthesia, flash frozen in dry ice and cut onto 10-µm-thick sections containing the amygdala. Before hybridization, sections were fixed (cold 4% PFA for 20 min), dehydrated in ethanol series, and treated with a protease (30 min RT), according to the manufacturers' protocol. The hybridization reaction included RNAscope Probes Rn-Pvalb-C3, Rn-Slc17a7-C2, and a custom-ordered Rn-Grik1-C1, targeting the same region of *Grik1* as the DIG-labeled probe [69]. The hybridization was done according to the protocols provided by the manufacturer. Stained sections were imaged with Zeiss Axio Imager.M2 microscope equipped with ApoTome 2 structured illumination slider, using PlanApo ×20 objective and CMOS camera (Hamamatsu ORCA Flash 4.0 V2) and Zen 2 software. The density of stained cells within a brain region of interest was calculated using ImageJ 1.52i software, for at least four sections/animal and three animals/group.

### Electrophysiology

#### Preparation of acute slices

Two- to three-month-old rats or mice were anesthetized with isoflurane and quickly decapitated. The brain was extracted and immediately placed in carbogenated (95% O_2_/5%CO_2_) ice-cold modified *N*-Methyl-d-glucamine (NMDG) based protective cutting solution (pH 7.3–7.4) containing (in mM): 92 NMDG, 2.5 KCl, 1.25 NaH_2_PO_4_, 30 NaHCO_3_, 20 HEPES, 25 glucose, 2 thiourea, 5 Na-ascorbate, 3 Na-pyruvate, 0.5 CaCl_2_·4H_2_O and 10 MgSO_4_·7H_2_O [70]. The cerebellum and a small part of the prefrontal cortex were trimmed off and the remaining block was glued to a stage and transferred to a vibratome (Leica VT 1200S) to obtain 350-µm-thick brain slices. Slices containing the amygdala were placed into a slice holder and incubated for 6–10 min in 34 °C in the NMDG–based solution. Slices were then transferred into a separate slice holder in room temperature with solution containing (in mM): 92 NaCl, 2.5 KCl, 1.25 NaH_2_PO_4_, 30 NaHCO_3_, 20 HEPES, 25 glucose, 2 thiourea, 5 Na-ascorbate, 3 Na-pyruvate, 2 CaCl_2_·4H_2_O and 2 MgSO_4_·7H_2_O (saturated with 95% O_2_/5%CO_2_).

#### Electrophysiological recordings

After 1–5 h of recovery, the slices were placed in a submerged heated (32–34 °C) recording chamber and perfused with standard ACSF containing (in mM): 124 NaCl, 3 KCl, 1.25 NaH_2_PO_4_, 26 NaHCO_3_, 15 glucose 1 MgSO_4_^.^ 7H_2_O, 2 CaCl_2_ at the speed of 1–2 ml/min. Whole-cell patch-clamp recordings were done from LA neurons under visual guidance using patch electrodes with resistance of 2–7 MΩ. Cells with capacitance >50 pF and spike frequency adaptation to membrane depolarization were considered principal neurons. Multiclamp 700B amplifier (Molecular Devices), Digidata 1322 (Molecular Devices) or NI USB-6341 A/D board (National Instruments) and WinLTP version 2.20 [71] or pClamp 11.0 software were used for data collection, with low pass filter (10 kHz) and sampling rate of 10–20 kHz. In all voltage-clamp recordings, uncompensated series resistance (Rs) was monitored by measuring the peak amplitude of the fast whole-cell capacitance current in response to a 5 mV step. Only experiments where Rs <30 MΩ, and with <20% change in Rs during the experiment, were included in analysis. The drugs were purchased from Hello Bio (Picrotoxin, d-(-)-2-amino-5-phosphonopentanoic acid (d-AP5), tetrodotoxin (TTX) and CNQX) or Tocris Bioscience (ACET and L-689,560).

*Glutamatergic responses (EPSCs)* were recorded from LA neurons at a holding potential of −70 mV using electrodes filled with the following solution (in mM): 130 CsMeSO_4_, 10 HEPES, 0.5 EGTA, 8 NaCl, 4 Mg-ATP, 0.3 Na-GTP, 5 QX314, 280 mOsm, pH 7.2. Picrotoxin (100 µM) and D-AP5 (50 µM) were included in the perfusion solution to antagonize fast GABA_A_ and NMDAR-mediated components of synaptic transmission, respectively. EPSCs were evoked with a bipolar stimulation electrodes (nickel-chromium wire), placed in the external and internal capsule to mimic activation of cortical and thalamic inputs, respectively. Paired-pulse responses (PPR) were evoked with inter-pulse interval of 50 ms.

*Spontaneous and evoked GABAergic responses (IPSCs)* were recorded from LA neurons using a cesium-based filling solution (pH 7.2, 280 mOsm) containing (in mM): 125.5 CsMeSO_4_, 10 HEPES, 5 EGTA, 8 NaCl, 4 Mg-ATP, 0.3 Na-GTP, 5 QX314. L-689,560 (5 µM) was included in the ACSF to prevent possible NMDAR dependent synaptic plasticity. For recording of mIPSC, also 1 µM TTX was added to the perfusion solution. The membrane potential was first clamped at −70 mV, and the cell was allowed to equilibrate with the filling solution for 5–10 min. In some experiments evoked EPSCs were collected at −70 mV, after which the membrane potential was slowly raised to 0 mV, the reversal potential of glutamatergic currents, in order to isolate fast GABA_A_-R mediated currents. In these experiments, the Vm values were corrected for the calculated LJP. The cell was allowed to stabilize at 0 mV for 5–10 min prior to baseline measurement.

For recording of monosynaptic IPSCs, a stimulation electrode was placed ~100 µm away from the recorded cell, in order to stimulate only the local inhibitory network surrounding it. Disynaptic IPSCs were evoked by stimulation of external and internal capsule. 10 µM CNQX was applied at the end of the experiments to ensure that the recorded IPSC was monosynaptic (i.e. no effect) or disynaptic (> 80% block of the IPSC amplitude). Paired-pulse responses were evoked with inter-pulse interval of 50 ms (SOM) or 100 ms (PNs).

*Drug-induced responses* were recorded from SOM+ PV+ and VIP+ neurons in the LA, in the presence of antagonists for AMPA, NMDA and GABAA receptors (2 µM NBQX, 5 µM L-689,560 and 100 µM picrotoxin, respectively) using intracellular solution containing (in mM): 135 K-gluconate, 10 HEPES, 5 EGTA, 2 KCl, 2 Ca(OH)_2_, 4 Mg-ATP, 0.5 Na-GTP, (280 mOsm, pH 7.2). Drugs (2 µM KA, 1 µM ATPA and 200 nM ACET, with 33 mM KCl as positive control) were diluted in ACSF and applied through valve control system (VC-6-PINCH, Warner Instruments) with a manifold.

*Whole-cell current-clamp recordings of intrinsic excitability* were performed using the above filling solution and drug application system, with unmodified ACSF. The cells were recorded in the resting membrane potential (PV, VIP) or at −80 mV (SOM). Input resistance and spike frequency were monitored by passing square-wave current pulses (amplitude, −50 pA–300 pA; duration, 400 ms).

*For whole-cell current-clamp recordings of evoked EPSPs*, patch electrodes (2–5 MΩ) contained the following (in mM): 130 K-gluconate, 10 HEPES, 0.5 EGTA, 7 NaCl, 4 Mg-ATP, 0.3 Na-GTP, 280 mOsm, pH 7.2. EPSPs were recorded at a membrane potential between −60 and −70 mV and in the presence of NMDAR antagonist L-689,560 (5 µM). EPSPs were evoked by stimulation of external and internal capsule using an intensity that resulted in action potential firing in approximately 50% of the trials.

*Data analysis*. WinLTP program was used to calculate the peak amplitude of the evoked synaptic responses. For PPR and EPSC/dIPSC ratios, 7–10 responses were averaged in each experimental condition. PPR was calculated as the amplitude ratio of response 2/response 1, and EPSC/dIPSC ratio as the amplitude ratio of response at -70 mV/response at 0 mV. The frequency and amplitude of spontaneous synaptic events were analyzed using Minianalysis program 6.0.3. sIPSCs and mIPSCs were identified in the analysis as outward currents with typical kinetics that were at least three times the amplitude of the baseline level of noise. For the time course plots all events are averaged with 1–2 min bins and normalized to the baseline before application of the drug. Averages for baseline, drug application, and washout were calculated over a 10 min period. Pooled data on the intrinsic firing frequency of the interneurons has been analyzed using step 100 pA from the rheobase. AP half-width and amplitude of the hyperpolarizing potential are analyzed from the 3^rd^ spike in the train using Clampfit software.

### Stereotaxic surgery and behavioral tests

AAV serotype 8 viral vectors encoding for GFP (pAAV.CMV.PI.EGFP.WPRE.bGH) and GFP-CRE (pAAV.CMV.HI.eGFP-Cre.WPRE.SV40) were purchased from Addgene (catalog #105530-AAV8 and 105545-AAV8, respectively). The AAV particles were bilaterally injected to the BLA of adult (>P55) *Grik1*^*tm1c*/*tm1c*^ mice under anesthesia in a stereotaxic frame as described [13] using the following coordinates: (1) from bregma AP −1,5 ML 3.4 DV 4.0–4.1 (2) from bregma AP −1,8 ML 3.4 DV 4.0–4.1. The surgery was carried out between P60-P75 and behavioral tests were started 4–5 weeks after the surgery (age 3–4 months). A separate group of mice was used for electrophysiological analysis 3–5 weeks after the AAV injections.

After the viral injection, the animals were individually housed to prevent fighting and avoid openings of stitches. Behavioral tests were performed between 9 am - 4 pm, during light period (lights on 6 a.m., off 6 p.m.). The stage of the estrus cycle was not taken into account. For all the behavioral tests, the investigator was blinded to the group allocation.

The location of the injection site was confirmed after completion of the battery of behavioral tests. Only mice with a strong GFP signal in the BLA were included in the analysis. The infection rate in different amygdala nuclei was quantified using slices from three animals that had passed the visual inspection criteria. The percentage of GFP positive neurons from all DAPI positive cells in LA, BA and CeA was calculated from Z-stack confocal images (8–12 images with interval of 10μm).

#### Elevated plus maze (EPM)

Mouse EPM consisted of two open arms (30 × 5 cm) and two enclosed arms (30 × 5 cm, inner diameter) connected by central platform (5 × 5 cm) and elevated to 40 cm above the floor. Open arms were surrounded by 0.5 cm high edges to prevent accidental falling of mice. The floor of each arm was light gray and the closed arms had transparent (15 cm high) side- and end-walls. The illumination level in all arms was ~150 lx. Rat EPM was made of dark gray PVC with two open arms (50 × 10 cm), two closed arms (50 ×10 c,) central platform (10 ×10 cm). Side and end-walls were non-transparent (40 cm high). Maze was elevated to 50 cm above the floor.

The animal was placed in the center of the maze facing one of the closed arms and observed for 5 min. The trials were recorded with Ethovision XT 10 videotracking software (Noldus Information Technology, Wageningen, The Netherlands). The latency to the first open-arm entry, number of open and closed arm entries (four paw criterion) and the time spent in different zones of the maze were measured. The number of fecal boli was counted after trial.

#### Open field (OF)

Activity Monitor (MedAssociates, St. Albans, VT) with infrared photo-beam tracking technology (sensors detecting horizontal and vertical activity) was used to measure the animal behavior in the OF. The size of the arena for mouse was 28 × 28 cm, and for rat 44 × 44 cm. The animal was released in the corner of arena and monitored for 30 min. The distance traveled and time spent in the center of the arena was measured.

### Statistical analysis

All statistical analyses were done on raw (not normalized) data using Sigma Plot 11.0 software. The sample size was based on previous experience on similar experiments. All data were first assessed for normality and homogeneity of variance and the statistical test was chosen accordingly. Differences between two groups were analyzed using two-tailed *t*-test or Mann–Whitney rank-sum test. Two-way ANOVA with Holm–Sidak post-hoc comparison was used to compare effects of gender and stress. For data that were not normally distributed, Mann–Whitney test was used for post-hoc pairwise comparison. To compare drug effects to the baseline, Student’s paired two-tailed *t*-test was used. The one-sample *t*-test was used to evaluate significance of drug-induced currents. The results were considered significant when *p* < 0.05. All the pooled data are given as mean ± SEM.

## Supplementary information

Supplementary Material

Supplementary Figure 1

Supplementary Figure 2

Supplementary Figure 3

Supplementary table 1

## References

[1] Danielewicz J, Hess G (2014). Early life stress alters synaptic modification range in the ratlateral amygdala. Behav Brain Res.

[2] Guadagno A, Wong TP, Walker CD (2018). Morphological and functional changes in thepreweaning basolateral amygdala induced by early chronic stress associate withanxiety and fear behavior in adult male, but not female rats. Prog NeuropsychopharmacolBiol Psychiatry.

[3] Raineki C, Opendak M, Sarro E, Showler A, Bui K, McEwen BS, (2019). During infantmaltreatment, stress targets hippocampus, but stress with mother present targetsamygdala and social behavior. Proc Natl Acad Sci USA.

[4] Krugers HJ, Arp JM, Xiong H, Kanatsou S, Lesuis SL, Korosi A (2016). Early life adversity: Lasting consequences for emotional learning. Neurobiol Stress.

[5] Murthy S, Gould E (2020). How early life adversity influences defensive circuitry. Trends Neurosci.

[6] VanTieghem MR, Tottenham N (2018). Neurobiological programming of early life stress: functional development of amygdala-prefrontal circuitry and vulnerability for stress-related psychopathology. Curr Top Behav Neurosci.

[7] Lerma J (2006). Kainate receptor physiology. Curr Opin Pharmacol.

[8] Shin RM, Tully K, Li Y, Cho JH, Higuchi M, Suhara T (2010). Hierarchical order of coexisting pre- and postsynaptic forms of long-term potentiation at synapses in amygdala. Proc Natl Acad Sci USA..

[9] Cho JH, Bayazitov IT, Meloni EG, Myers KM, Carlezon WA, Zakharenko SS (2011). Coactivation of thalamic and cortical pathways induces input timing-dependent plasticity in amygdala. Nat Neurosci.

[10] Braga MF, Aroniadou-Anderjaska V, Xie J, Li H (2003). Bidirectional modulation of GABA release by presynaptic glutamate receptor 5 kainate receptors in the basolateral amygdala. J Neurosci.

[11] Wu LJ, Ko SW, Toyoda H, Zhao MG, Xu H, Vadakkan KI (2007). Increased anxiety-like behavior and enhanced synaptic efficacy in the amygdala of GluR5 knockout mice. PLoS ONE.

[12] Arora V, Pecoraro V, Aller MI, Román C, Paternain AV, Lerma J (2018). Increased Grik4 gene dosage causes imbalanced circuit output and human disease-related behaviors. Cell Rep.

[13] Ryazantseva M, Englund J, Shintyapina A, Huupponen J, Shteinikov V, Pitkänen A (2020). Kainate receptors regulate development of glutamatergic synaptic circuitry in the rodent amygdala. Elife.

[14] Aller MI, Pecoraro V, Paternain AV, Canals S, Lerma J (2015). Increased dosage of high-affinity kainate receptor gene grik4 alters synaptic transmission and reproduces autism spectrum disorders features. J Neurosci.

[15] Alt A, Weiss B, Ornstein PL, Gleason SD, Bleakman D, Stratford RE (2007). Anxiolytic-like effects through a GLUK5 kainate receptor mechanism. Neuropharmacology.

[16] Ko S, Zhao MG, Toyoda H, Qiu CS, Zhuo M (2005). Altered behavioral responses to noxious stimuli and fear in glutamate receptor 5 (GluR5)- or GluR6-deficient mice. J Neurosci.

[17] Läck AK, Ariwodola OJ, Chappell AM, Weiner JL, McCool BA (2008). Ethanol inhibition of kainate receptor-mediated excitatory neurotransmission in the rat basolateral nucleus of the amygdala. Neuropharmacology..

[18] Masneuf S, Lowery-Gionta E, Colacicco G, Pleil KE, Li C, Crowley N (2014). Glutamatergic mechanisms associated with stress-induced amygdala excitability and anxiety-related behavior. Neuropharmacology.

[19] Douglas LN, McGuire AB, Manzardo AM, Butler MG (2016). High-resolution chromosome ideogram representation of recognized genes for bipolar disorder. Gene.

[20] Gray AL, Hyde TM, Deep-Soboslay A, Kleinman JE, Sodhi MS (2015). Sex differences in glutamate receptor gene expression in major depression and suicide. Mol Psychiatry.

[21] Le-Niculescu H, Patel SD, Bhat M, Kuczenski R, Faraone SV, Tsuang MT (2009). Convergent functional genomics of genome-wide association data for bipolar disorder: comprehensive identification of candidate genes, pathways and mechanisms. Am J Med Genet B Neuropsychiatr Genet..

[22] Paddock S, Laje G, Charney D, Rush AJ, Wilson AF, Sorant AJ (2007). Association of GRIK4 with outcome of antidepressant treatment in the STAR*D cohort. Am J Psychiatry.

[23] Scarr E, Beneyto M, Meador-Woodruff JH, Dean B (2005). Cortical glutamatergic markers in schizophrenia. Neuropsychopharmacology.

[24] Lerma J, Marques JM (2013). Kainate receptors in health and disease. Neuron.

[25] Li H, Rogawski MA (1998). GluR5 kainate receptor mediated synaptic transmission in rat basolateral amygdala in vitro. Neuropharmacology.

[26] Li H, Chen A, Xing G, Wei ML, Rogawski MA (2001). Kainate receptor-mediated heterosynaptic facilitation in the amygdala. Nat Neurosci.

[27] Shin RM, Higuchi M, Suhara T (2013). Nitric oxide signaling exerts bidirectional effects on plasticity inductions in amygdala. PLoS ONE.

[28] Dargan SL, Clarke VR, Alushin GM, Sherwood JL, Nisticò R, Bortolotto ZA (2009). ACET is a highly potent and specific kainate receptor antagonist: characterisation and effects on hippocampal mossy fibre function. Neuropharmacology.

[29] Wyeth MS, Pelkey KA, Yuan X, Vargish G, Johnston AD, Hunt S (2017). Neto auxiliary subunits regulate interneuron somatodendritic and presynaptic kainate receptors to control network inhibition. Cell Rep.

[30] Daw MI, Pelkey KA, Chittajallu R, McBain CJ (2010). Presynaptic kainate receptor activation preserves asynchronous GABA release despite the reduction in synchronous release from hippocampal cholecystokinin interneurons. J Neurosci.

[31] Lourenço J, Cannich A, Carta M, Coussen F, Mulle C, Marsicano G (2010). Synaptic activation of kainate receptors gates presynaptic CB(1) signaling at GABAergic synapses. Nat Neurosci..

[32] Huntley MA, Srinivasan K, Friedman BA, Wang TM, Yee AX, Wang Y (2020). Genome-wide analysis of differential gene expression and splicing in excitatory neurons and interneuron subtypes. J Neurosci.

[33] Valbuena S, Lerma J (2016). Non-canonical signaling, the hidden life of ligand-gated ion channels. Neuron.

[34] Wolff SB, Gründemann J, Tovote P, Krabbe S, Jacobson GA, Müller C (2014). Amygdala interneuron subtypes control fear learning through disinhibition. Nature.

[35] Prager EM, Bergstrom HC, Wynn GH, Braga MF (2016). The basolateral amygdala γ-aminobutyric acidergic system in health and disease. J Neurosci Res.

[36] Sharp BM (2017). Basolateral amygdala and stress-induced hyperexcitability affect motivated behaviors and addiction. Transl Psychiatry.

[37] Babaev O, PIletti Chatain C, Krueger-Burg D (2018). Inhibition in the amygdala anxiety circuitry. Exp Mol Med.

[38] Zhang X, Ge TT, Yin G, Cui R, Zhao G, Yang W (2018). Stress-induced functional alterations in amygdala: implications for neuropsychiatric diseases. Front Neurosci.

[39] Lee SC, Amir A, Haufler D, Pare D (2017). Differential recruitment of competing valence-related amygdala networks during anxiety. Neuron.

[40] Zhang JY, Liu TH, He Y, Pan HQ, Zhang WH, Yin XP (2019). Chronic stress remodels synapses in an amygdala circuit-specific manner. Biol Psychiatry.

[41] Akgül G, McBain CJ (2016). Diverse roles for ionotropic glutamate receptors on inhibitory interneurons in developing and adult brain. J Physiol.

[42] Contractor A, Mulle C, Swanson GT (2011). Kainate receptors coming of age: milestones of two decades of research. Trends Neurosci.

[43] Krabbe S, Gründemann J, Lüthi A (2018). Amygdala inhibitory circuits regulate associative fear conditioning. Biol Psychiatry.

[44] Krabbe S, Paradiso E, d'Aquin S, Bitterman Y, Courtin J, Xu C (2019). Adaptive disinhibitory gating by VIP interneurons permits associative learning. Nat Neurosci.

[45] Rhomberg T, Rovira-Esteban L, Vikór A, Paradiso E, Kremser C, Nagy-Pál P (2018). Vasoactive intestinal polypeptide-immunoreactive interneurons within circuits of the mouse basolateral amygdala. J Neurosci.

[46] Hu H, Gan J, Jonas P (2014). Interneurons. Fast-spiking, parvalbumin(+) GABAergic interneurons: from cellular design to microcircuit function. Science.

[47] Veres JM, Nagy GA, Hájos N (2017). Perisomatic GABAergic synapses of basket cells effectively control principal neuron activity in amygdala networks. eLife.

[48] Hale MW, Johnson PL, Westerman AM, Abrams JK, Shekhar A, Lowry CA (2010). Multiple anxiogenic drugs recruit a parvalbumin-containing subpopulation of GABAergic interneurons in the basolateral amygdala. Prog Neuro-Psychopharmacol Biol Psychiatry.

[49] Lukkes JL, Burke AR, Zelin NS, Hale MW, Lowry CA (2012). Post-weaning social isolation attenuates c-Fos expression in GABAergic interneurons in the basolateral amygdala of adult female rats. Physiol Behav..

[50] Butler RK, White LC, Frederick-Duus D, Kaigler KF, Fadel JR, Wilson MA (2012). Comparison of the activation of somatostatin- and neuropeptide Y-containing neuronal populations of the rat amygdala following two different anxiogenic stressors. Exp Neurol.

[51] Zhang W, Rosenkranz JA (2016). Effects of repeated stress on age-dependent GABAergic regulation of the lateral nucleus of the amygdala. Neuropsychopharmacology..

[52] Giachino C, Canalia N, Capone F, Fasolo A, Alleva E, Riva MA (2007). Maternal deprivation and early handling affect density of calcium binding protein-containing neurons in selected brain regions and emotional behavior in periadolescent rats. Neuroscience.

[53] Gildawie KR, Honeycutt JA, Brenhouse HC (2020). Region-specific effects of maternal separation on perineuronal net and parvalbumin-expressing interneuron formation in male and female rats. Neuroscience.

[54] Santiago AN, Lim KY, Opendak M, Sullivan RM, Aoki C (2018). Early life trauma increases threat response of peri-weaning rats, reduction of axo-somatic synapses formed by parvalbumin cells and perineuronal net in the basolateral nucleus of amygdala. J Comp Neurol.

[55] Seidel K, Helmeke C, Poeggel G, Braun K (2008). Repeated neonatal separation stress alters the composition of neurochemically characterized interneuron subpopulations in the rodent dentate gyrus and basolateral amygdala. Dev Neurobiol.

[56] Luo ZY, Huang L, Lin S, Yin YN, Jie W, Hu NY (2020). Erbin in amygdala parvalbumin-positive neurons modulates anxiety-like behaviors. Biol Psychiatry.

[57] Robinson SL, Thiele TE (2020). A role for the neuropeptide somatostatin in the neurobiology of behaviors associated with substances abuse and affective disorders. Neuropharmacology..

[58] Bissière S, Humeau Y, Lüthi A (2003). Dopamine gates LTP induction in lateral amygdala by suppressing feedforward inhibition. Nat Neurosci.

[59] Higley MJ (2014). Localized GABAergic inhibition of dendritic Ca(2+) signalling. Nat Rev Neurosci.

[60] Ito W, Fusco B, Morozov A (2020). Disinhibition-assisted long-term potentiation in the prefrontal-amygdala pathway via suppression of somatostatin-expressing interneurons. Neurophotonics.

[61] Valbuena S, García Á, Mazier W, Paternain AV, Lerma J (2019). Unbalanced dendritic inhibition of CA1 neurons drives spatial-memory deficits in the Ts2Cje Down syndrome model. Nat Commun.

[62] Zhuo M (2017). Cortical kainate receptors and behavioral anxiety. Mol Brain.

[63] Orav E, Atanasova T, Shintyapina A, Kesaf S, Kokko M, Partanen J (2017). NETO1 guides development of glutamatergic connectivity in the hippocampus by regulating axonal kainate receptors. eNeuro.

[64] Dehorter N, Ciceri G, Bartolini G, Lim L, del Pino I, Marin O (2015). Tuning of fast-spiking interneuron properties by an activity-dependent transcriptional switch. Science.

[65] Miller MN, Okaty BW, Kato S, Nelson SB (2011). Activity-dependent changes in the firing properties of neocortical fast-spiking interneurons in the absence of large changes in gene expression. Dev Neurobiol.

[66] Bartley AF, Huang ZJ, Huber KM, Gibson JR (2008). Differential activity-dependent, homeostatic plasticity of two neocortical inhibitory circuits. J Neurophysiol.

[67] Bath KG (2020). Synthesizing views to understand sex differences in response to early life adversity. Trends Neurosci.

[68] Blume SR, Freedberg M, Vantrease JE, Chan R, Padival M, Record MJ (2017). Sex- and estrus-dependent differences in rat basolateral amygdala. J Neurosci.

[69] Vesikansa A, Sakha P, Kuja-Panula J, Molchanova S, Rivera C, Huttunen HJ (2012). Expression of GluK1c underlies the developmental switch in presynaptic kainate receptor function. Sci Rep.

[70] Ting JT, Daigle TL, Chen Q, Feng G (2014). Acute brain slice methods for adult and aging animals: application of targeted patch clamp analysis and optogenetics. Methods Mol Biol..

[71] Anderson WW, Collingridge GL (2007). Capabilities of the WinLTP data acquisition program extending beyond basic LTP experimental functions. J Neurosci Methods.

