# Supplementary material for: Downregulation of kainate receptors regulating GABAergic transmission in amygdala after early life stress is associated with anxiety-like behavior in rodents
Source: Transl Psychiatry. 2021 Oct 18;11:538. doi: 10.1038/s41398-021-01654-7 (PMC8523542; doi:10.1038/s41398-021-01654-7)
Supplement: Supplementary file 1 — Supplementary Material [file 41398_2021_1654_MOESM1_ESM.docx]

**Supplementary material**

**Supplementary Figure 1.** Cell-type specific expression pattern of Grik1 varies between brain regions.

1. Triple-ISH staining for *Grik1* (white*)*, *PValb* (parvalbumin, green) and *Slc17a7* (VGlut1, red) in different regions of the adult rat hippocampus (male). *Grik1* is expressed in *Slc17a7*-negative, non-glutamatergic neurons and in a subpopulation *PValb-*positive neurons (yellow arrows).
2. Example of the triple-ISH staining for *Grik1* (white*)*, *PValb* (parvalbumin, green) and *Slc17a7* (VGlut1, red) in the retrosplenial cortex (RSC), parietal associational cortex (PtA) and ventral auditory cortex (AuV). Prominent co-localization of *Grik1* and *Slc17a7* (red arrows) is seen in RSC and in AuV, while in PtA *Grik1* co-localizes with certain *PVAlb* neurons.

**
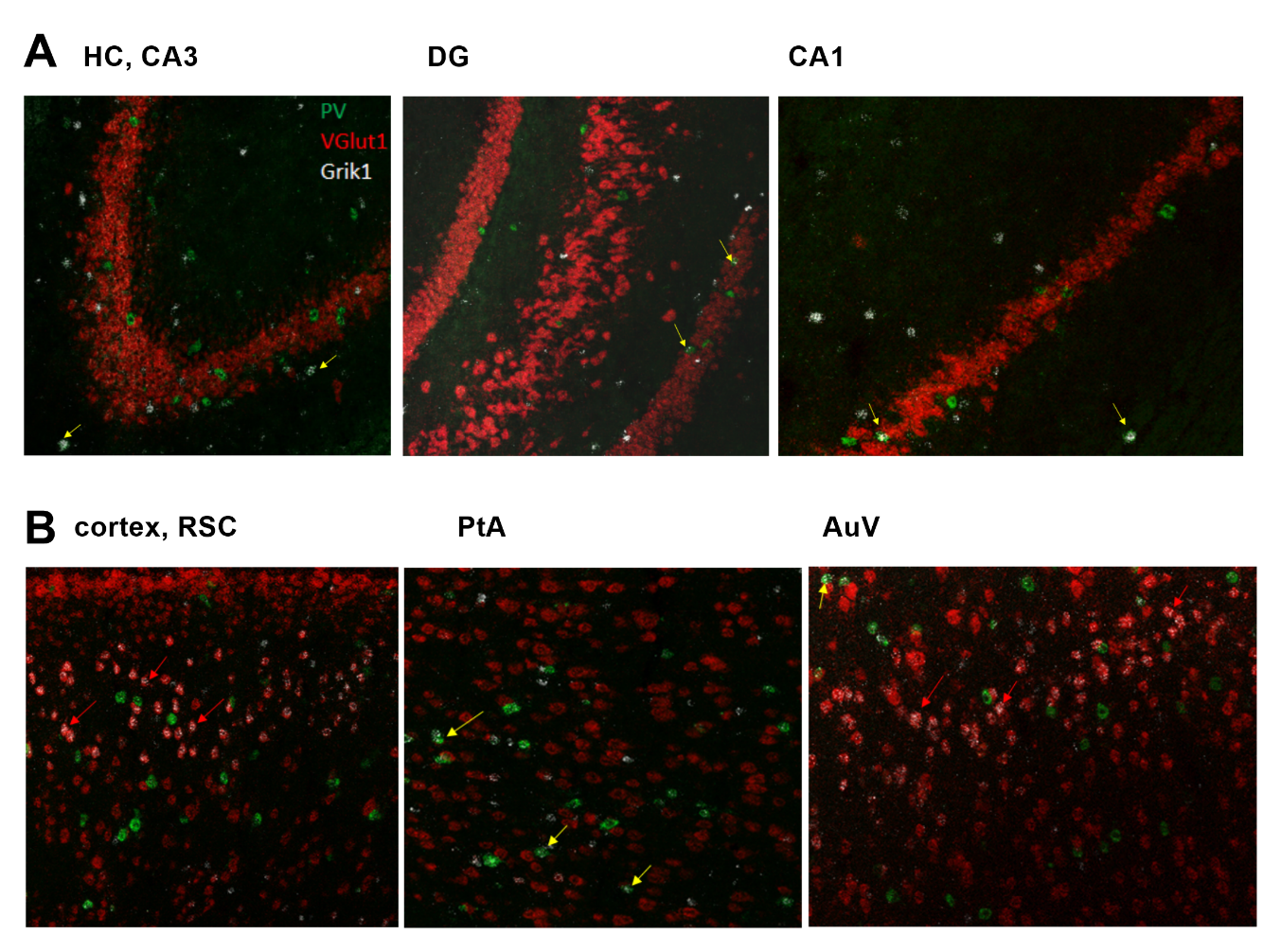
**

**Supplementary Figure 2**. MS does not significantly influence KAR dependent modulation of glutamatergic inputs to LA PNs.

1. Example traces and pooled data on the effect of GluK1 antagonist ACET (200 – 500 nM) on excitatory postsynaptic currents (EPSCs), evoked by electrical stimulation of cortical afferents and recorded from LA PNs, in control (n=12) and MS (n=11) rats. The traces represent examples from the time points indicated (1-3) in the time-course plot. The EPSC amplitude in ACET, normalized to the baseline level in individual experiments in male and female rats in both groups (control M, 0.95 ± 0.04, n=6; control F, 0.80 ± 0.03, n=6; MS M, 0.94 ± 0.08, n=6; MS F, 0.96 ± 0.06, n=5) * p=0.004, paired t-test.
2. Effect of ACET on paired-pulse ratio (PPR) of evoked EPSCs in cortical inputs in female control and MS rats. Example traces and pooled data, representing PPR in ACET normalized to the baseline level in individual experiments (control, 1.25 ± 0.07; MS 0.99 ± 0.07, n=5 in both groups), * p< 0.05, paired t-test.
3. Similar data as in A, for EPSCs evoked by stimulation of thalamic afferents to LA (control M, 0.99 ± 0.04, n=8; control F, 0.98 ± 0.08, n=5; MS M, 1.04 ± 0.14, n=5; MS F, 0.97 ± 0.04, n=6).


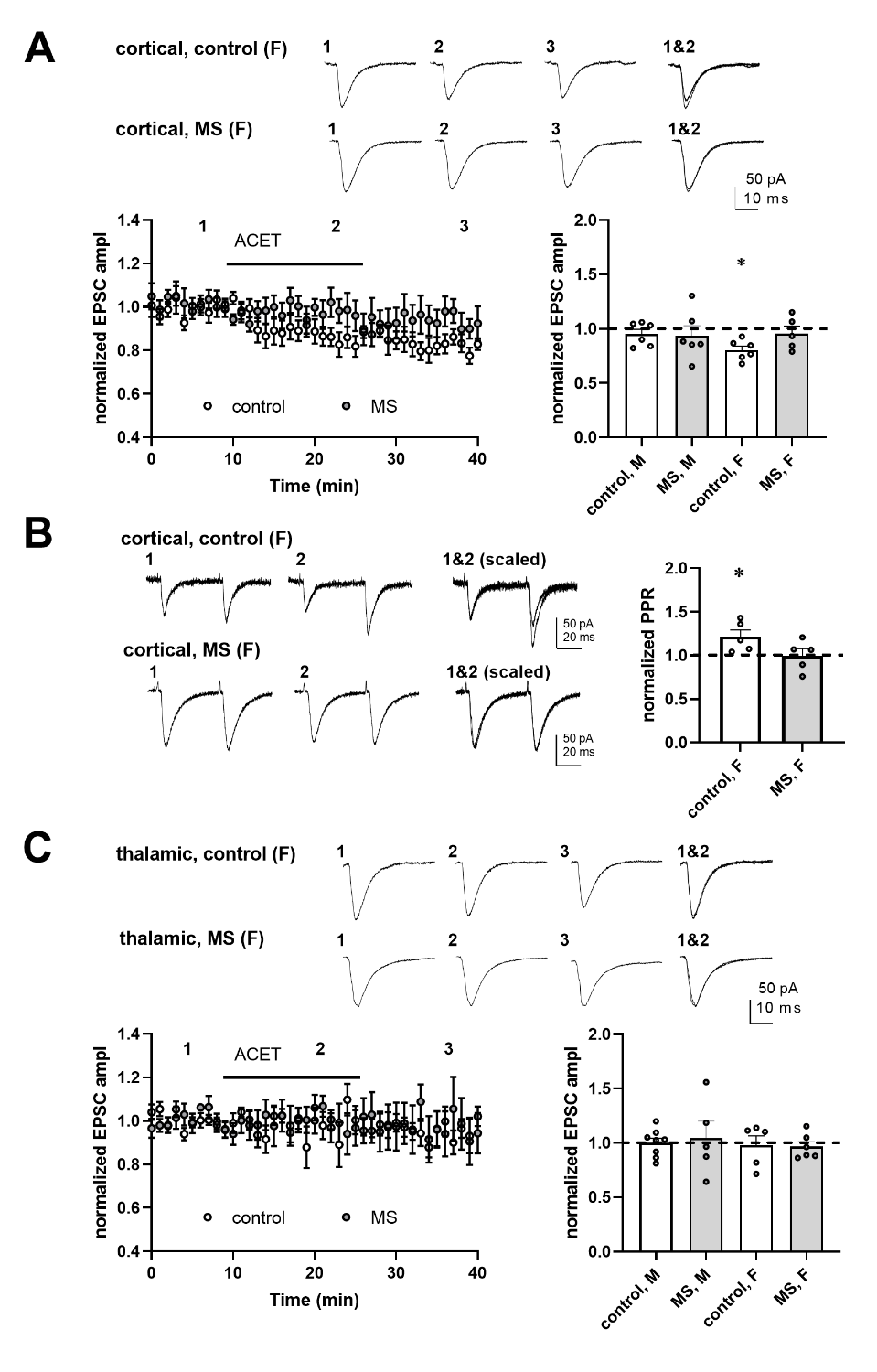


**Supplementary Figure 3.** KAR antagonism has no consistent effect on GABA release to LA PNs

1. Effect of ACET (200 nM) on the frequency of mIPSCs in LA PNs in control male rats. Example traces and mean mIPSC frequency in individual cells in baseline, during ACET application and after ACET washout (n=6). Pooled data on the effect of ACET on fast (rise time ≤ 2ms) and slow (rise time > 2ms) mIPSC events in individual cells.
2. Effect of ACET (200 nM) on evoked monosynaptic IPSCs (eIPSCs) in LA PNs in control male rats (n=8). Example traces and time course plot depicting the normalized amplitude of IPSCs in baseline, ACET and after ACET washout. CNQX was applied at the end of the experiment to assure monosynaptic nature of the responses. The graphs on the bottom row show the effect of ACET on first IPSC amplitude, normalized to the baseline level (1.10 ± 0.06) and PPR (1.07 ± 0.16), and a plot of the change in IPSC amplitude against PPR in individual cells.


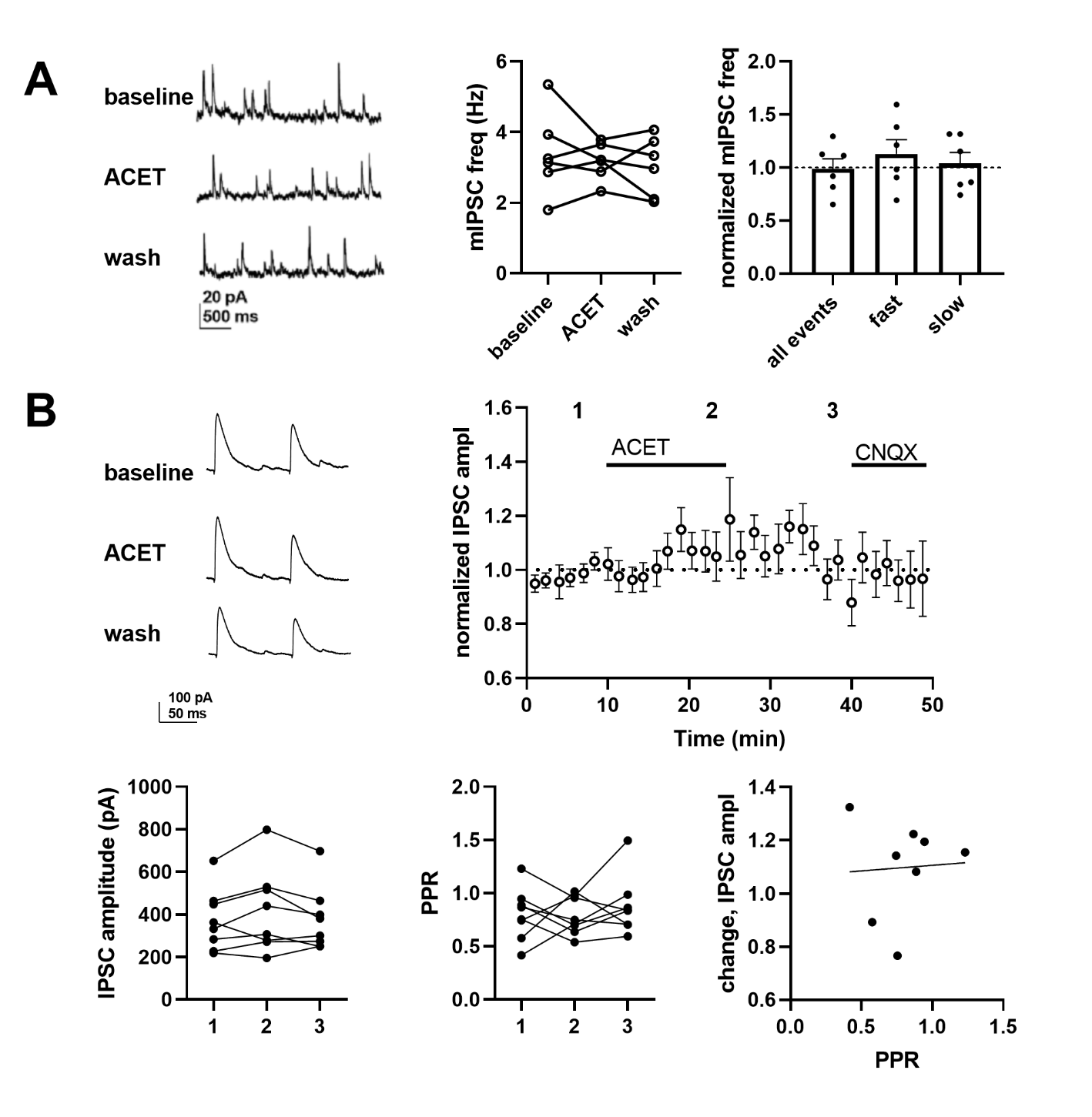


**Supplementary table 1.**

Values for sIPSC frequency and amplitude, for the data shown in Figure 3A

|  | **Basal** | | **Effect of ACET (normalized to baseline)** | |
| --- | --- | --- | --- | --- |
|  | **sIPSC freq (Hz)** | **sIPSC ampl (pA)** | **sIPSC freq** | **sIPSC ampl** |
| control, M | 13.4 ± 1.7 | 19.7 ± 1.6 | 1.51 ± 0.2 * | 1.01 ± 0.04 |
| MS , M | 16.4 ± 1.2 | 17.2 ± 0.9 | 0.86 ± 0.05 * | 1.02 ± 0.04 |
| control, F | 17.5 ± 2.1 | 19.3 ± 1.7 | 0.95 ± 0.07 | 1.01 ± 0.03 |
| MS, F | 14.7 ± 2.5 | 18.1 ± 1.6 | 1.01 ± 0.13 | 1.03 ± 0.07 |

Values for mIPSC frequency and amplitude, for the data shown in Supplementary Figure 3A

|  | **Basal** | | **Effect of ACET (normalized to baseline)** | |
| --- | --- | --- | --- | --- |
|  | **mIPSC freq (Hz)** | **mIPSC ampl (pA)** | **mIPSC freq** | **mIPSC ampl** |
| control, M | 3.2 ± 0.45 | 17.1 ± 0.9 | 1.09 ± 0.12 | 1.00 ± 0.03 |

Values for sIPSC frequency and amplitude, for the data shown in Figure 6A and B

|  | **Basal** | | **Effect of ACET (normalized to baseline)** | |
| --- | --- | --- | --- | --- |
|  | **sIPSC freq (Hz)** | **sIPSC ampl (pA)** | **sIPSC freq** | **sIPSC ampl** |
| SOM | - 1. ± 0.88 | 32.2 ± 8.0 | 0.48 ± 0.09 | 1.01 ± 0.05 |
| PV | 10.0 ± 3.3 | 25.9 ± 0.8 | 0.98 ± 0.02 | 0.95 ± 0.05 |

Values for mIPSC frequency and amplitude, for the data shown in Figure 6C

|  | **Basal** | | **Effect of ACET (normalized to baseline)** | |
| --- | --- | --- | --- | --- |
|  | **mIPSC freq (Hz)** | **mIPSC ampl (pA)** | **mIPSC freq** | **mIPSC ampl** |
| SOM | 3.2 ± 0.74 | 20.5 ± 1.1 | 1.0 ± 0.09 | 1.02 ± 0.03 |
