# Supplementary figures and images for: Downregulation of kainate receptors regulating GABAergic transmission in amygdala after early life stress is associated with anxiety-like behavior in rodents
Source: Transl Psychiatry. 2021 Oct 18;11:538. doi: 10.1038/s41398-021-01654-7 (PMC8523542; doi:10.1038/s41398-021-01654-7)

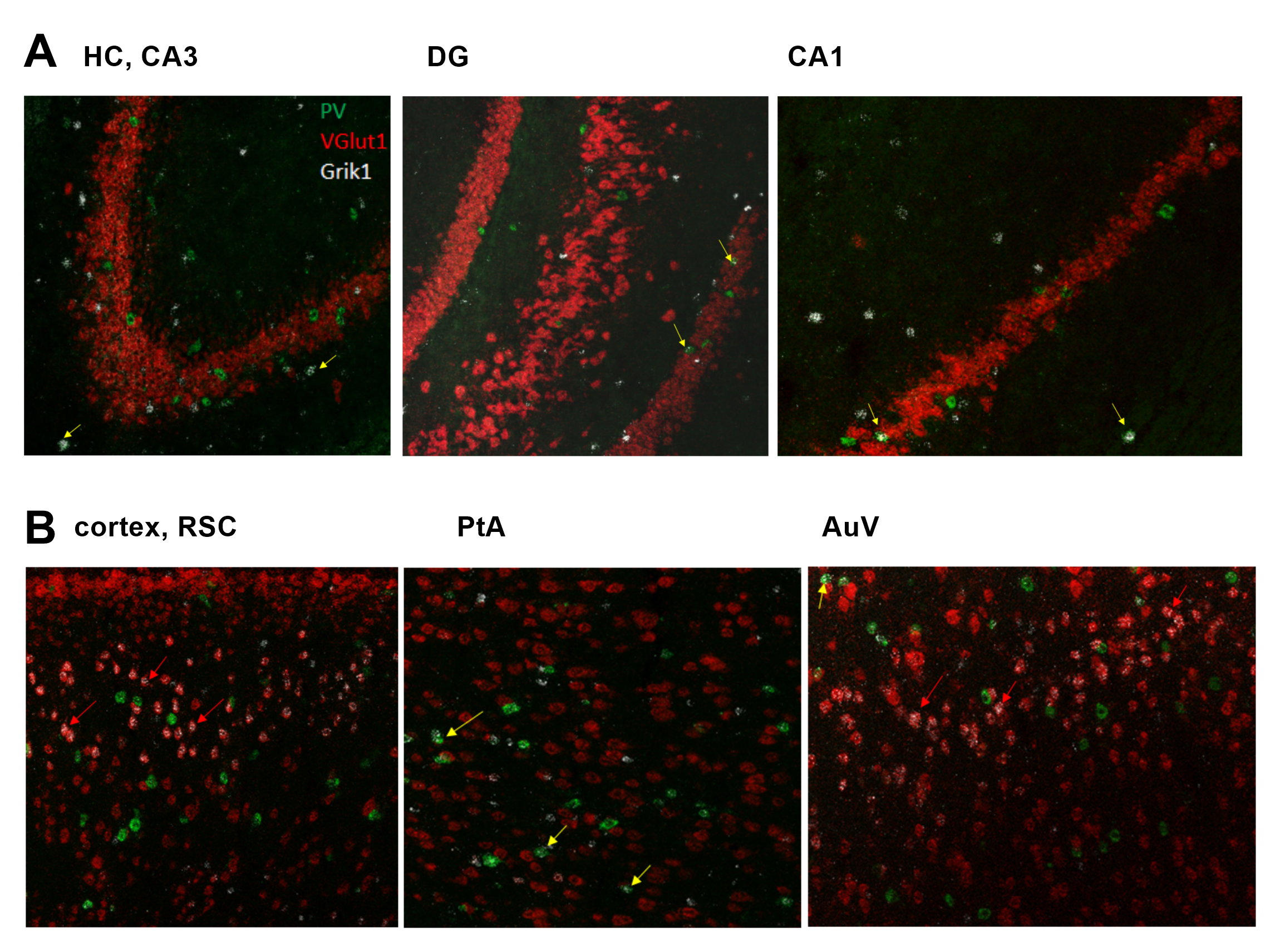

Supplement: Supplementary file 2 — Supplementary Figure 1 [file 41398_2021_1654_MOESM2_ESM.tif]

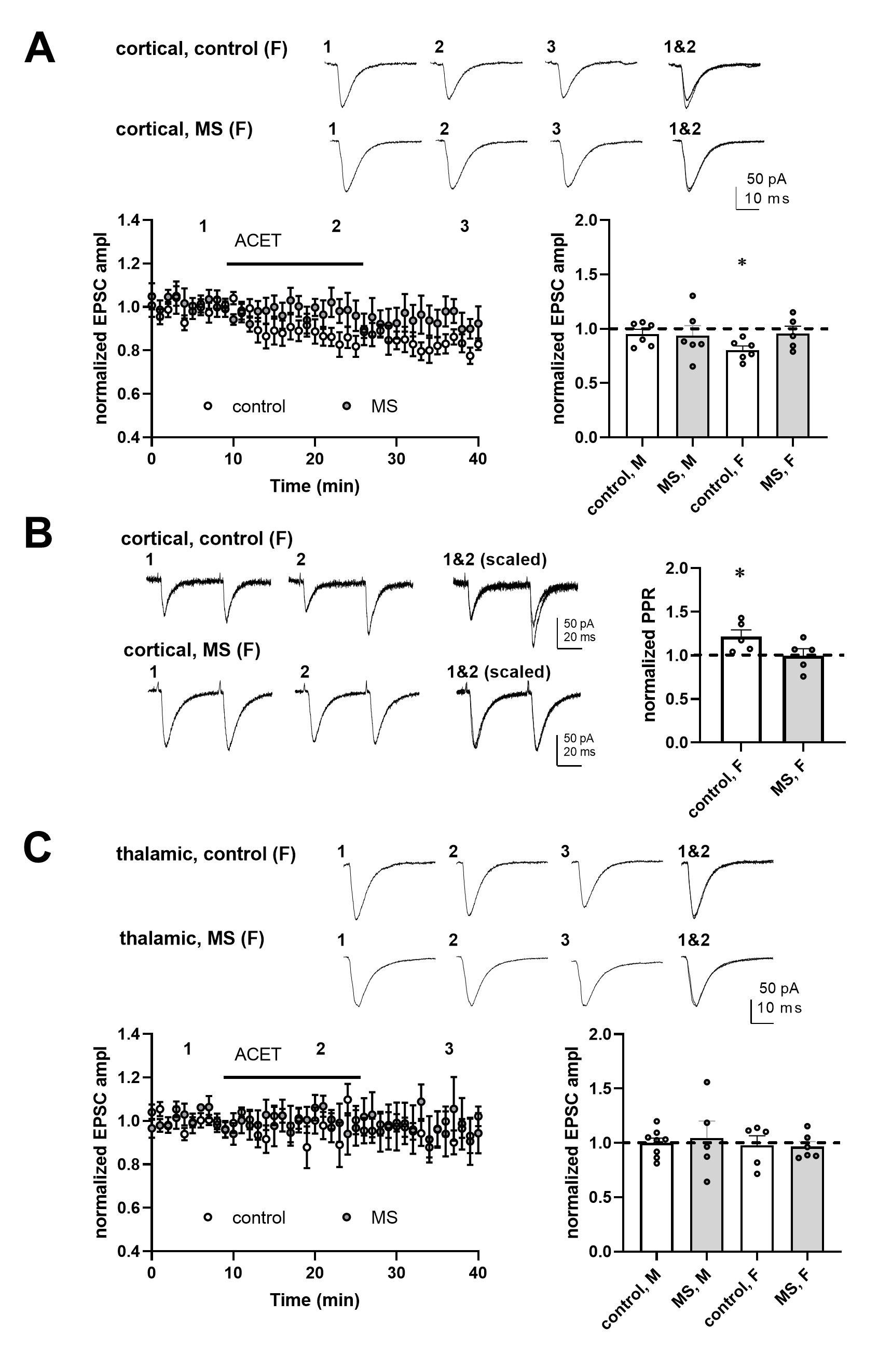

Supplement: Supplementary file 3 — Supplementary Figure 2 [file 41398_2021_1654_MOESM3_ESM.tif]

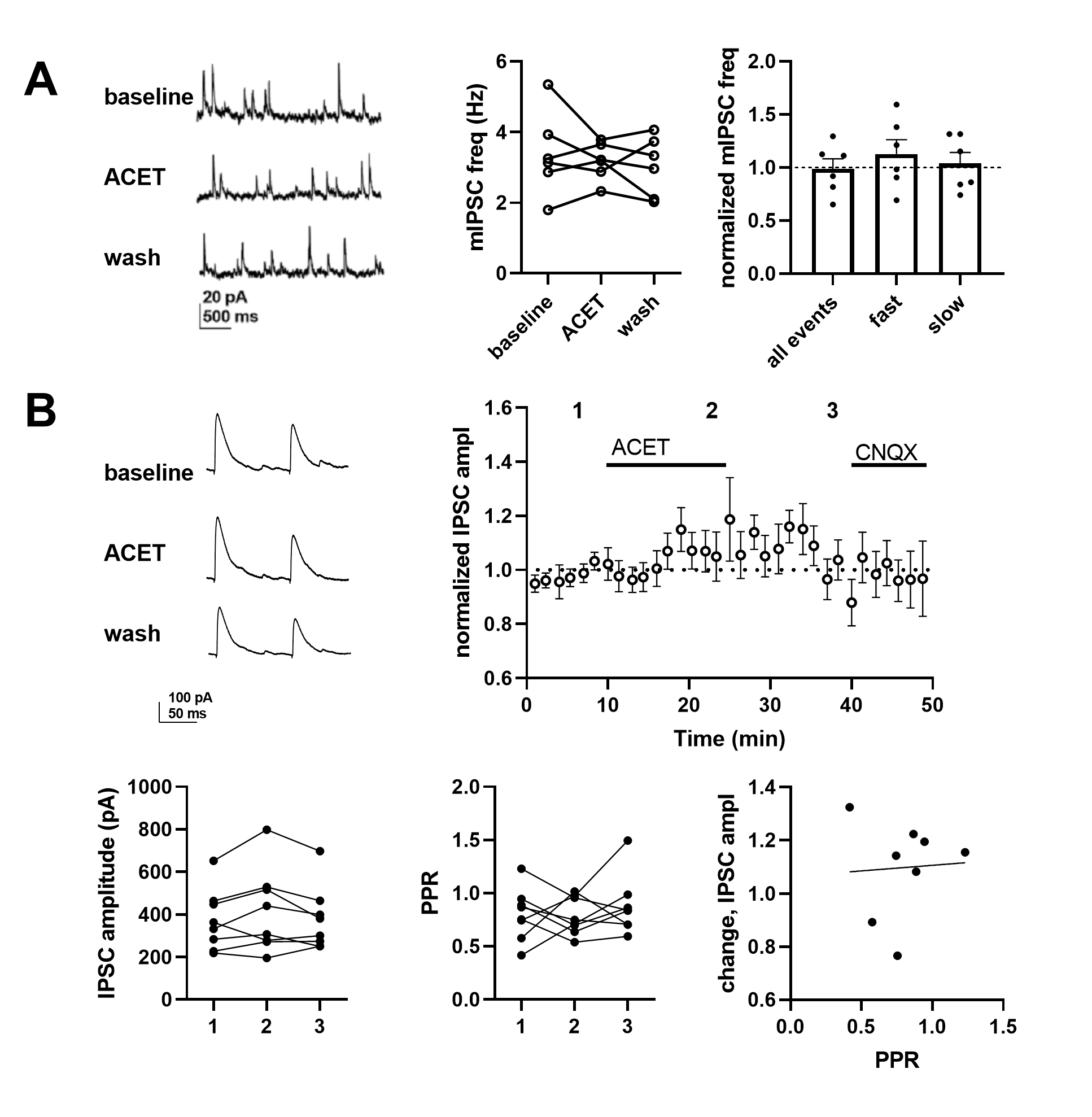

Supplement: Supplementary file 4 — Supplementary Figure 3 [file 41398_2021_1654_MOESM4_ESM.tif]
