# Supplementary material for: Downregulation of kainate receptors regulating GABAergic transmission in amygdala after early life stress is associated with anxiety-like behavior in rodents
Source: Transl Psychiatry. 2021 Oct 18;11:538. doi: 10.1038/s41398-021-01654-7 (PMC8523542; doi:10.1038/s41398-021-01654-7)
Supplement: Supplementary file 5 — Supplementary table 1 [file 41398_2021_1654_MOESM5_ESM.docx]

**Supplementary table 1.**

Values for sIPSC frequency and amplitude, for the data shown in Figure 3A

|  | **Basal** | | **Effect of ACET (normalized to baseline)** | |
| --- | --- | --- | --- | --- |
|  | **sIPSC freq (Hz)** | **sIPSC ampl (pA)** | **sIPSC freq** | **sIPSC ampl** |
| control, M | 13.4 ± 1.7 | 19.7 ± 1.6 | 1.51 ± 0.2 * | 1.01 ± 0.04 |
| MS , M | 16.4 ± 1.2 | 17.2 ± 0.9 | 0.86 ± 0.05 * | 1.02 ± 0.04 |
| control, F | 17.5 ± 2.1 | 19.3 ± 1.7 | 0.95 ± 0.07 | 1.01 ± 0.03 |
| MS, F | 14.7 ± 2.5 | 18.1 ± 1.6 | 1.01 ± 0.13 | 1.03 ± 0.07 |

Values for mIPSC frequency and amplitude, for the data shown in Supplementary Figure 3A

|  | **Basal** | | **Effect of ACET (normalized to baseline)** | |
| --- | --- | --- | --- | --- |
|  | **mIPSC freq (Hz)** | **mIPSC ampl (pA)** | **mIPSC freq** | **mIPSC ampl** |
| control, M | 3.2 ± 0.45 | 17.1 ± 0.9 | 1.09 ± 0.12 | 1.00 ± 0.03 |

Values for sIPSC frequency and amplitude, for the data shown in Figure 6A and B

|  | **Basal** | | **Effect of ACET (normalized to baseline)** | |
| --- | --- | --- | --- | --- |
|  | **sIPSC freq (Hz)** | **sIPSC ampl (pA)** | **sIPSC freq** | **sIPSC ampl** |
| SOM | - 1. ± 0.88 | 32.2 ± 8.0 | 0.48 ± 0.09 | 1.01 ± 0.05 |
| PV | 10.0 ± 3.3 | 25.9 ± 0.8 | 0.98 ± 0.02 | 0.95 ± 0.05 |

Values for mIPSC frequency and amplitude, for the data shown in Figure 6C

|  | **Basal** | | **Effect of ACET (normalized to baseline)** | |
| --- | --- | --- | --- | --- |
|  | **mIPSC freq (Hz)** | **mIPSC ampl (pA)** | **mIPSC freq** | **mIPSC ampl** |
| SOM | 3.2 ± 0.74 | 20.5 ± 1.1 | 1.0 ± 0.09 | 1.02 ± 0.03 |
